# Investigation of the evolution of Pd-Pt supported on ceria for dry and wet methane oxidation

**DOI:** 10.1038/s41467-022-32765-4

**Published:** 2022-08-29

**Authors:** Núria. J. Divins, Andrea Braga, Xavier Vendrell, Isabel Serrano, Xènia Garcia, Lluís Soler, Ilaria Lucentini, Maila Danielis, Andrea Mussio, Sara Colussi, Ignacio J. Villar-Garcia, Carlos Escudero, Alessandro Trovarelli, Jordi Llorca

**Affiliations:** 1 Institute of Energy Technologies, Universitat Politècnica de Catalunya, EEBE, Eduard Maristany 10-14, 08019 Barcelona, Spain; 2 Department of Chemical Engineering, Universitat Politècnica de Catalunya, EEBE, Eduard Maristany 10-14, 08019 Barcelona, Spain; 3 Barcelona Research Center in Multiscale Science and Engineering, Universitat Politècnica de Catalunya, EEBE, Eduard Maristany 10-14, 08019 Barcelona, Spain; 4 Dipartimento Politecnico, Università di Udine, and INSTM, Via del Cotonificio 108, 33100 Udine, Italy; 5 ALBA Synchrotron Light Source, Carrer de la Llum 2-26, 08290 Cerdanyola del Vallès, Barcelona, Spain

**Keywords:** Heterogeneous catalysis, Catalyst synthesis, Catalytic mechanisms

## Abstract

Efficiently treating methane emissions in transportation remains a challenge. Here, we investigate palladium and platinum mono- and bimetallic ceria-supported catalysts synthesized by mechanical milling and by traditional impregnation for methane total oxidation under dry and wet conditions, reproducing those present in the exhaust of natural gas vehicles. By applying a toolkit of in situ synchrotron techniques (X-ray diffraction, X-ray absorption and ambient pressure photoelectron spectroscopies), together with transmission electron microscopy, we show that the synthesis method greatly influences the interaction and structure at the nanoscale. Our results reveal that the components of milled catalysts have a higher ability to transform metallic Pd into Pd oxide species strongly interacting with the support, and achieve a modulated PdO/Pd ratio than traditionally-synthesized catalysts. We demonstrate that the unique structures attained by milling are key for the catalytic activity and correlate with higher methane conversion and longer stability in the wet feed.

## Abstract (web summary)

Methane is a strong contributor to global warming. Here the authors demonstrate that mechanically prepared Pd-Pt supported on ceria catalysts abate methane better than conventionally impregnated ones, even under wet conditions, owing to a highly dynamic structure.

## Introduction

Palladium-platinum-based materials, alone or in combination with Rh, are the state-of-the-art catalysts for different after-treatment systems of the exhausts from mobile sources^1–4^. Bimetallic formulations containing Pd and Pt are used on natural-gas-fueled vehicles (NGVs) for the abatement of unburned methane^5–7^, which is a powerful greenhouse gas with a global warming potential 86 times that of CO_2_ on a 20-year period, and 34 times higher on a 100-year horizon^8^. Due to the exponential growth of NGVs in the last few years, boosted also by the increase in the renewable natural gas-fueled vehicles^9,10^, addressing and decreasing methane emissions to the atmosphere has become of increasing concern and the optimization of the catalytic system has attracted growing interest^7,11,12^. The addition of platinum to Pd-based catalysts, which are widely recognized as the most active for methane oxidation, has proved to be effective in improving the catalyst stability against sulfur poisoning^13–15^ and deactivation induced by the large steam content present in the exhausts^16–19^.

In the last few years, many efforts have been carried out to identify the role of platinum on catalyst activity and stability. The vast majority of results have been obtained by using ex situ tools. Nevertheless, identifying the actual working active sites and understanding their evolution requires in situ and operando techniques^20,21^. In this regard, by using in situ X-ray absorption fine structure spectroscopy (XAFS), it has been observed that Pd oxidation is a prerequisite to CH_4_ combustion activity^22^. The improved activity of bimetallic Pd-Pt catalysts in wet conditions was paralleled with a lack of surface oxygen, which did not occur under a dry methane-lean feed, where oxygen poisoned Pt^23^. Under low temperature lean CH_4_ combustion conditions, it was also found that while a monometallic Pd catalyst was fully oxidized between 473–773 K, a Pd-Pt (Pd:Pt ratio 2:1) catalyst showed the coexistence of Pd and PdO under the same temperature range. This indicated that Pt promoted the formation of a reduced Pd phase, which was considered less active than PdO for methane combustion^24^. By in situ XAFS, the evolution of a bimetallic PtPd catalyst was followed, assessing its higher stability against sintering compared to a Pd-only sample thanks to the formation of a core-shell structure^25^. A recent near ambient pressure X-ray photoelectron spectroscopy (AP-XPS) study has shown that, under lean conditions, Pd tends to be fully oxidized making less clear the contribution of platinum on the catalytic activity in comparison to the behavior under stoichiometric conditions, where the bimetallic catalyst shows lower light-off temperature ascribed to the coexistence of Pd^2+^ and Pd^0^ promoted by the presence of Pt^26^. Previously, by in situ XPS, it was determined that the surface fraction of Pd^2+^ depended on Pt content^27^. Despite some apparent contradiction in the results, which are likely due to different experimental conditions, Pd:Pt ratios and oxygen content, all these works agree on the strong effect of platinum on Pd electronic state and, consequently, on catalytic properties.

All the above-mentioned in situ studies refer to Pd-Pt catalysts supported on alumina. However, a key factor affecting the activity of Pd-based catalysts is the support and, among the oxides tested, CeO_2_ is known to play a crucial role in enhancing the catalytic activity and stability for methane oxidation^28^. Moreover, the support also has a great influence in the rearrangement of bimetallic nanoparticles, and to track successfully their reorganization in situ and operando techniques are required^21,29^. Additionally, the synthesis method has been demonstrated to have an enormous impact on the catalytic performance as well^30–33^. In this regard, by mechanochemical milling, which is a simple, eco-friendly (no solvents are used) method, and readily transferrable to industry, enhanced activity and stability of a PtPd/CeO_2_ catalyst has been reported compared to a monometallic Pd/CeO_2_ and the same sample prepared by traditional incipient wetness impregnation tested in wet lean methane oxidation^34^.

In this work, we investigate a series of mono- and bimetallic Pd and Pt CeO_2_-supported catalysts prepared via mechanical milling with a toolkit of in situ techniques, including synchrotron X-ray diffraction, XAFS, and AP-XPS, and high-resolution transmission electron microscopy (HRTEM) observations. By combining the results of these techniques, we provide fundamental insights into the metal-ceria interface, the impact of adding Pt on its evolution, the structure and the surface of methane combustion catalysts under relevant NGVs conditions, i.e., under the presence of steam and low temperatures, conditions required to meet future regulations. In situ studies and HRTEM observations reveal the presence of a highly dynamic structure in the mechanically synthesized catalysts with a modulated Pd/PdO ratio, undergoing a distinctive reorganization under reaction conditions, and we ultimately correlate this information with the catalytic performance.

## Results and discussion

### Catalysts synthesis and activation

The mechanochemical synthesis is a facile, eco-friendly, and readily scalable method where no solvents are used to prepare the catalysts^32^. Four catalysts were synthesized by mechanical milling (denoted as MM): (1) a monometallic 1.5 wt% Pd-CeO_2_ catalyst; (2) a monometallic 1.5 wt% Pt-CeO_2_ catalyst; (3) a PtPd-CeO_2_ catalyst where 1.5 wt% Pd was first milled with CeO_2_ and this was subsequently milled with 1.5 wt% Pt; and (4) a PdPt-CeO_2_ catalyst where the milling order was inverted (1.5 wt% Pt was first milled with CeO_2_ and next 1.5 wt% Pd was added and subsequently milled; note the difference in the order of the metals in the nomenclature)^34^. Additionally, a PdPt/CeO_2_ catalyst prepared by traditional incipient wetness impregnation (denoted as IWI) was also synthesized as reference material (see Methods), where Pd and Pt were co-impregnated, and it was calcined at 1173 K.

Initially, an aging treatment under dry lean methane combustion conditions up to the maximum temperatures present at the exhaust of NGVs was performed^11^. The treatment consisted of a temperature-programmed combustion (TPC) cycle up to 1173 K (denoted as TPC1173 treatment) under a dry lean methane mixture (0.5% CH_4_, 2% O_2_, balanced in He, GHSV = 2 × 10^5^ h^−1^). At 1173 K, the temperature was held for 1 min and then the catalysts were cooled down to room temperature (RT) (Fig. S1a).

### Evolution of the crystalline structure during dry methane combustion

The crystalline structure of the samples and their evolution under reaction conditions (see the “Methods” section for details) was investigated by in situ SXRD. Table S1 lists the crystallite size obtained with the Scherrer Eq. (1) for the as-prepared catalysts and after the TPC1173 treatment.

**Fig. 1:**
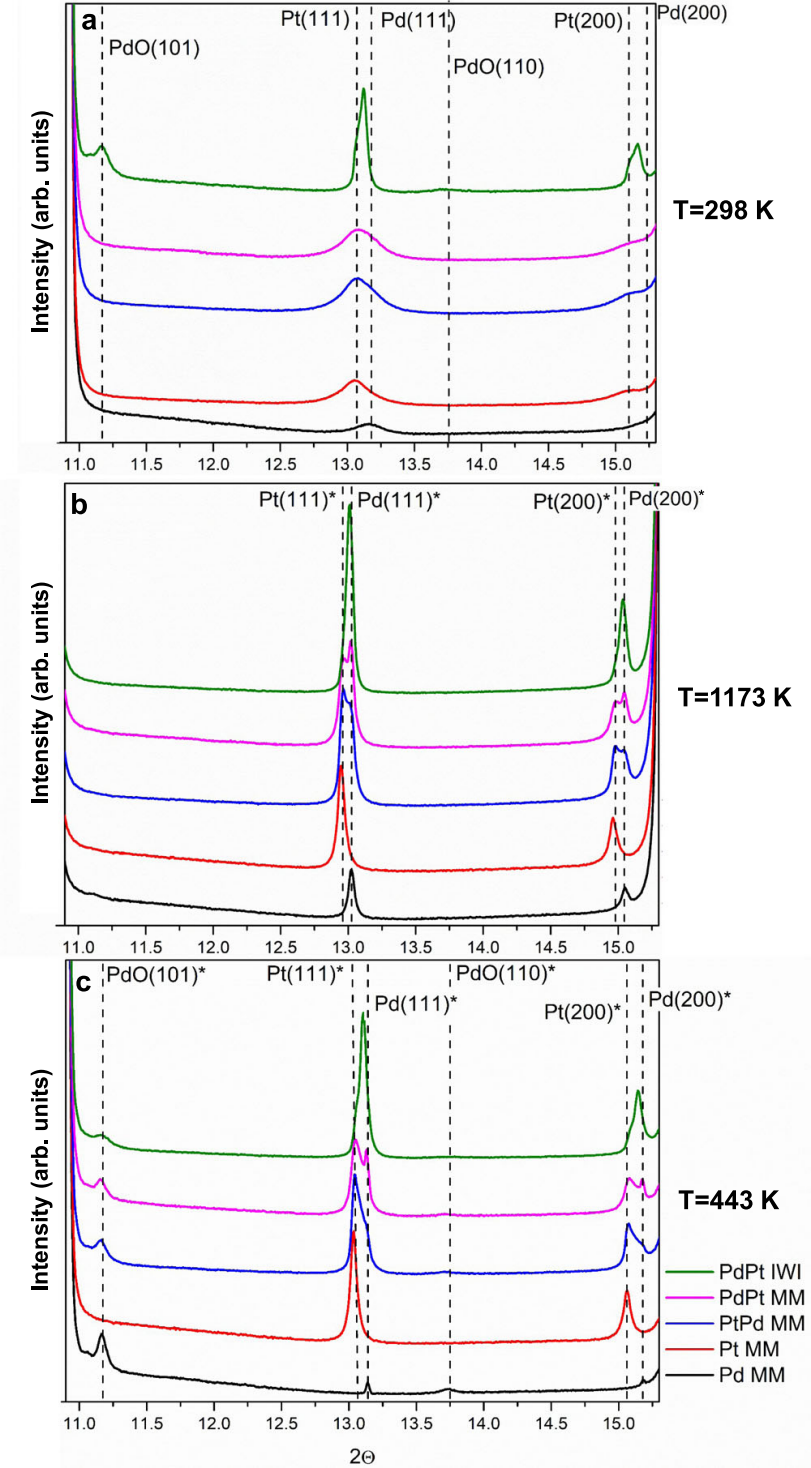
Diffractograms acquired under TPC conditions (CH_4_:O_2_:He = 0.5:2:97.5). **a** At room temperature, **b** 1173 K and **c** 443 K (during cooling down). The position of the peaks marked with * is shifted due to temperature (*λ* = 0.5157 Å). MM mechanical milling, IWI incipient wetness impregnation.

Initially, the structural reorganization undergone during the TPC1173 treatment was monitored. Figure 1 shows the diffractograms of the five catalysts investigated under TPC conditions at RT (fresh samples), at 1173 K, and at 443 K during the cooling down ramp (see Figs. S2 for the series of diffractograms and S3 for the evolution of the normalized weight fraction during ramping up and cooling down for sample Pd-CeO_2_ MM). The fresh MM catalysts show only broad peaks, indicating the presence of small metallic Pd and Pt nanoparticles (NPs) (Fig. 1a). PdO can only be detected for the PdPt/CeO_2_ IWI catalyst, as it was already calcined. Clearly, during the heating ramp, the NPs become larger and more crystalline (Fig. 1b). Interestingly, the phases formed at 1173 K depend on the synthesis method, MM vs. IWI, and the order of the milling process. In the metallic Pd and Pt regions, the milled bimetallic samples show two peaks, being more prominent the peak that corresponds to the last metal added to the milling process. Conversely, the IWI bimetallic catalyst shows a single asymmetric peak. During the cooling down ramp, the phases reorganize (Figs. 1c and S2). At 443 K, the Pd-CeO_2_ MM catalyst shows the least crystalline Pd phase together with the highest contribution of PdO. On the other hand, the Pt-CeO_2_ MM and the bimetallic catalysts show a higher degree of crystallinity. The PdPt/CeO_2_ IWI catalyst shows an asymmetric peak, probably indicating the formation of a PdPt alloy, as the position of this peak is in between the position of that for Pd(111) and Pt(111), as expected for an alloy. The asymmetry of the peak probably is related to some metallic Pd and/or Pt that remain segregated. Regarding the PdO concentration, the MM catalysts contain larger amounts than the IWI.

### Evolution of the crystalline structure during wet methane combustion

**Fig. 2:**
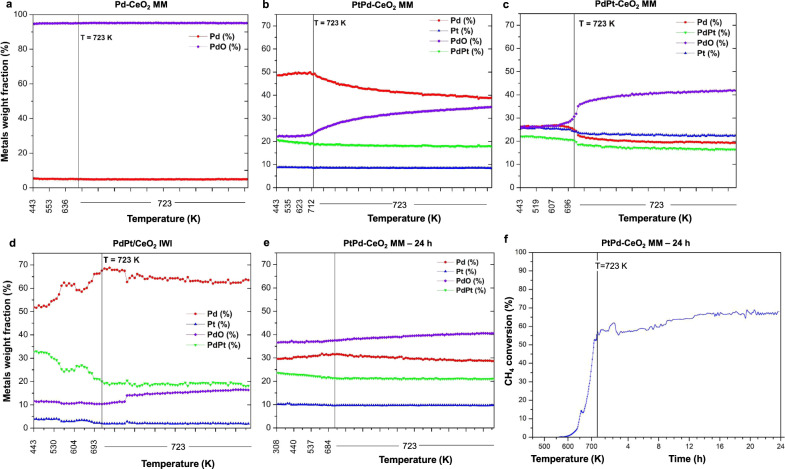
Evolution of the normalized weight fraction of the phases obtained from the Rietveld refinement under wet lean methane oxidation conditions at different temperatures. **a**–**e** For the indicated catalysts. **f** Methane conversion of PtPd-CeO_2_ MM under time-on-stream at 723 K for 24 h under wet lean methane conditions (0.5% CH_4_, 2% O_2_, 10% H_2_O, balance He). MM mechanical milling, IWI incipient wetness impregnation.

After the TPC1173 cycle, the wet lean methane combustion mixture, where 10% water vapor was present (0.5% CH_4_, 2% O_2_, 10% H_2_O, balance He), was dosed. Under the wet mixture, the samples were heated up from 443 to 723 K (10 K min^−1^), where the temperature was held for 2 h to track the evolution of the phases under NGVs operation. Figure 2 shows the evolution of the weight fraction obtained from the Rietveld refinement under wet methane combustion conditions (the series of diffractograms are shown in Figs. S4 and S5). The monometallic Pd-CeO_2_ MM catalyst shows the largest fraction of PdO (ca. 95 wt%) among all samples, which remains constant during the 2 h under wet conditions (Fig. 2a), and metallic Pd as a minority phase. In the monometallic Pt-CeO_2_ MM, only metallic Pt is detected and no hints of Pt oxides are observed (Fig. S6). Interestingly, the two bimetallic milled samples clearly show different phases. At the beginning of the wet experiments at 443 K, PtPd-CeO_2_ MM is composed mainly of metallic Pd and, in lower amounts, of PdO, PdPt alloy, and Pt (Fig. 2b). During the 2 h under wet reaction conditions at 723 K, the amount of PdO monotonically increased at the expense of metallic Pd, indicating that the presence of the wet mixture gradually oxidizes metallic Pd to form PdO. Conversely, the bimetallic PdPt-CeO_2_ MM catalyst initially shows a lower metallic Pd weight fraction than PtPd-CeO_2_ MM and a larger concentration of Pt, being the weight fractions of PdO, Pd, and Pt similar (Fig. 2c). For this catalyst, the oxidation of Pd to PdO occurs more abruptly than for PtPd-CeO_2_ MM, and the PdO concentration increases to a lesser extent over time-on-stream (TOS).

To further investigate the gradual increase of PdO for PtPd-CeO_2_ MM, which was the best performing catalyst in the wet atmosphere among the ones considered^34^, a PtPd-CeO_2_ MM catalyst treated in the laboratory for 24 h under TOS wet at 723 K was studied under in situ conditions. The in situ measurements started directly dosing the wet reaction mixture at RT and ramping it to 723 K (10 K min^–1^). This experiment also mimics a start-stop operation of the duty cycle. As seen in Fig. 2e, at this stage of the reaction, the PdO concentration keeps its ascending trend, while the amount of metallic Pd continues decreasing. Remarkably, a catalytic activity test was carried out for 24 h under wet conditions in the laboratory and not only a stable performance was obtained but a steady increase of the methane conversion during the 24 h under wet conditions over TOS was recorded (Fig. 2f), demonstrating that the milled catalyst does not deactivate in the presence of steam. This is in accordance with the catalytic performance reported in our previous work, where the sample milled with metallic Pd and, subsequently, metallic Pt exhibited simultaneously improved catalytic activity and stability under prolonged reaction exposure^34^. Here, in situ SXRD analysis further reveals that the increase in methane conversion parallels the increase of PdO concentration, thus allowing to correlate the progressive formation of PdO with the improvement of the catalytic performance. This result has been previously observed also for PtPd-alumina systems^22,35,36^. However, on these PtPd-ceria samples, it should be highlighted that, according to the most recent literature findings, the catalyst which shows the highest activity and stability, i.e., PtPd-CeO_2_ MM, is the one for which the PdO/Pd ratio becomes close to unity (Fig. 2b), and remains as such (Fig. 2e) during TOS, clearly indicating that not only the increase in PdO phase but also a suitable PdO/Pd ratio are necessary to achieve active and stable catalytic performances^37–39^. This is also confirmed by the different catalytic performances achieved by PtPd-CeO_2_ MM (most active and stable catalyst) and PdPt-CeO_2_ MM, i.e., the sample with inverse milling order.

Evident differences are observed between the IWI (Fig. 2d) and the MM catalysts. Initially, the PdPt/CeO_2_ IWI catalyst is composed of mostly metallic Pd and a larger fraction of PdPt alloy than the MM catalysts. Most importantly, for the IWI sample, the amount of metallic Pd becomes higher as the temperature under wet reaction conditions increases, which is the opposite trend observed for MM catalysts.

The evolution of the lattice parameters for the five catalysts investigated is displayed in Figs. S7 and S8. For none of the catalysts, distortion of the CeO_2_ or metals lattice was observed and only thermal expansion due to temperature could be detected.

### Evolution of the electronic structure during dry methane combustion

To gain information about the interaction between Pd, Pt, and Ce, on the most active and representative catalysts, namely Pd-CeO_2_ MM and PtPd-CeO_2_ MM^34^, the local electronic environment and structure of Pd K-edge and Pt L_3_-edge were investigated by in situ XAS under NGVs working conditions (see Methods) and compared with that of the IWI sample.

**Fig. 3:**
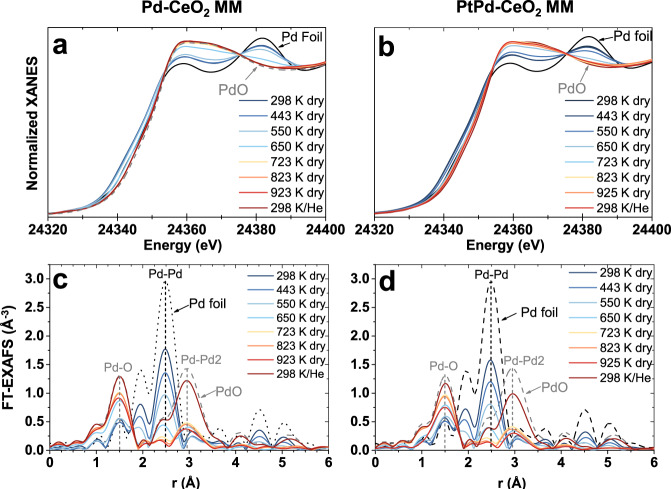
Series of in situ Pd K-edge data recorded under dry methane combustion conditions at increasing temperatures. **a**, **b** XANES spectra of Pd-CeO_2_ MM and PtPd-CeO_2_ MM, respectively. **c**, **d** Magnitude of the FT of the EXAFS spectra of Pd-CeO_2_ MM and PtPd-CeO_2_ MM catalysts. The spectra labeled as 298 K/He were acquired after the reaction series at room temperature under He. Reference spectra for a Pd foil and a PdO reference are also shown. MM mechanical milling.

Figure 3a, b shows the Pd K-edge X-ray absorption near-edge structure (XANES) spectra of as-prepared Pd-CeO_2_ MM and PtPd-CeO_2_ MM, respectively, during the TPC1173 treatment. Initially, the fresh monometallic sample is composed of a mixture of reduced and oxidized Pd species, which gradually oxidizes as the reaction temperature increases and exhibits a local structure similar to PdO, in accordance with the SXRD results outlined above. The magnitude of the Fourier transform (FT) of the extended X-ray absorption fine-structure (EXAFS) data is displayed in Fig. 3c, d. They show the coexistence of Pd-Pd bonds from metallic Pd and Pd-O bonds in the fresh catalysts and a gradual increase of the Pd-O bonds contribution, paralleling the decrease of Pd-Pd contribution, at increasing reaction temperature^40^. The FT-EXAFS data of the fresh Pd-CeO_2_ MM reveals that Pd is very well dispersed as the coordination number (CN) for the first Pd-Pd coordination shell is 7.5 ± 0.6, which corresponds to NPs of ca. 1 nm^41^, and the CN for the Pd-O bonds is 0.7 ± 0.2 (Table S2 and Fig. S10). A similar Pd local structure is found in PtPd-CeO_2_ MM, with a first shell Pd-M CN (M = Pd, Pt) of 6.9 ± 0.4 and Pd-O CN of 1.0 ± 0.2. At 650 K, Pd is almost completely oxidized in sample Pd-CeO_2_ MM and a similar trend is observed for PtPd-CeO_2_ MM. At temperatures above 650 K, no Pd-Pd bonds are detected for any of the samples, indicating that the catalysts are fully oxidized above this temperature. After the TPC measurements, the catalysts were cooled down in He to RT and spectra were collected, where a more developed PdO structure is detected for the monometallic catalyst.

**Fig. 4:**
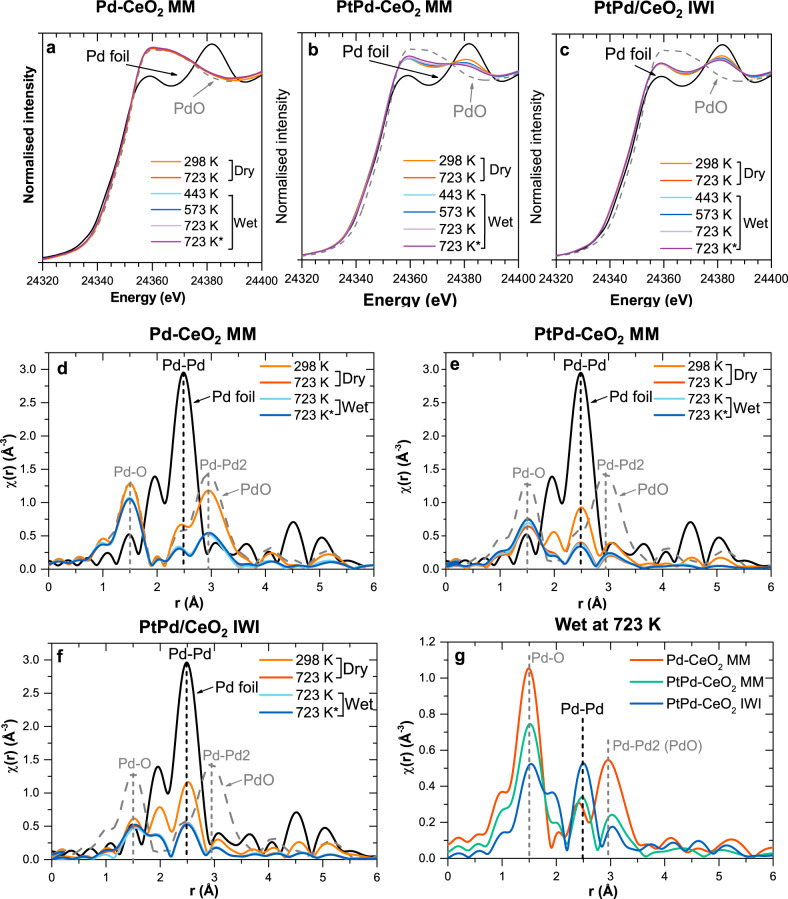
In situ Pd K-edge XANES and EXAFS data. XANES spectra of **a** Pd-CeO_2_ MM, **b** PtPd-CeO_2_ MM, and **c** PdPt/CeO_2_ IWI after the TPC1173 treatment under dry and wet lean methane combustion conditions. The state at the beginning of the wet measurements at 723 K and after 2 h are shown, the latter marked with *. Magnitude of the FT of the EXAFS spectra of **d** Pd-CeO_2_ MM, **e** PtPd-CeO_2_ MM, and **f** PdPt/CeO_2_ IWI after the TPC1173 treatment under dry and wet lean methane combustion conditions at 723 K. The state at the beginning of the wet measurements at 723 K and after 2 h are shown, the latter marked with *. **g** overlay of the magnitude of the FT of the EXAFS spectra of the three samples after 2 h under the wet methane combustion atmosphere at 723 K. Reference data of a Pd foil and a PdO standard are also shown. MM mechanical milling, IWI incipient wetness impregnation.

Samples that were subjected to a TPC1173 treatment in the laboratory were also studied (Fig. S1b). After the TPC1173, evident differences are already visible at the Pd K-edge at RT (Fig. 4). The XANES spectrum of the monometallic Pd-CeO_2_ MM catalyst shows that Pd species are oxidized and exhibit a local structure similar to PdO (Fig. 4a). Conversely, the spectra of the MM and IWI bimetallic catalysts show a combination of PdO and metallic Pd, indicating that the addition of Pt to Pd-CeO_2_ catalysts results in a more reduced state of Pd, in agreement with the SXRD results and previous reports^23^. Interestingly, the XANES spectra of the milled PtPd-CeO_2_ MM present a higher contribution of PdO species than the IWI counterpart (compare Fig. 4b, c). Under in situ conditions at 723 K, only minor oxidation is observed in the XANES spectra for the three catalysts.

The EXAFS data acquired at RT for the TPC1173 samples confirmed that the monometallic Pd-CeO_2_ MM is heavily oxidized and revealed that it is the only sample showing a clearly developed shell at ca. 3.0 Å (uncorrected for phase shift), corresponding to Pd-Pd1 and Pd-Pd2 bonds of the PdO structure (Fig. 4d). These results indicate that during the TPC1173, Pd strongly oxidized and transformed to bulk PdO. On the other hand, both bimetallic catalysts (MM and IWI) present Pd-O bonds at ca. 1.5 Å, but with a lower contribution than for the monometallic catalyst (see Supplementary Information, SI). Upon exposure to the dry reaction mixture at different temperatures (only data at 723 K is shown in Fig. 4 for clarity), the bimetallic catalysts undergo oxidation, as deduced from the decrease of the Pd-M (M = Pd, Pt) features. In the MM catalyst, the Pd-M features almost disappear, being also the Pd-O bonds more prominent than for the IWI catalyst. Noteworthy, the IWI catalyst shows the highest contribution of Pd-M features and the lowest contribution for Pd-O, confirming that it is the most reduced catalyst (compare Fig. 4e, f).

### Evolution of the electronic structure during wet methane combustion

After the lean methane oxidation in situ experiments, the temperature was lowered to 443 K and steam was added to the reactant mixture. In Fig. 4, data under in situ conditions obtained at the beginning of the reaction under the wet mixture at 723 K and after 2 h are displayed. The monometallic catalyst barely changes during this period, remaining oxidized during the whole measurement. PtPd-CeO_2_ MM oxidizes, as its Pd-M bonds slightly decrease, whilst the contribution of Pd-O bonds undergoes a mild increase during operation under wet conditions. In contrast, for the IWI catalyst, only subtle changes are detected. These results are in good agreement with the bulk data obtained from in situ SXRD. In Fig. 4g, the EXAFS data of the three catalysts after 2 h under the wet mixture at 723 K are overlaid. Distinctively, the monometallic Pd-CeO_2_ MM is the most oxidized sample, showing a strong development of the PdO structure and a low contribution of Pd-Pd bonds. The comparison of the bimetallic catalysts reveals that the mechanochemically-synthesized catalyst shows higher Pd-O and Pd-Pd2 contributions from the PdO structure and a lower Pd-M feature than the IWI counterpart. During the in situ series almost no changes are observed on the Pt L_3_-edge XAS data (see Fig. S9).

### Evolution of the surface active sites

The outermost layers of catalysts are key to the catalysis phenomenon. Therefore, to understand the origin of the higher activity and stability of the milled catalysts, the surface active sites were probed by synchrotron AP-XPS under the dry and wet mixtures. Combining the AP-XPS results with the results of in situ SXRD and XAS allows obtaining a full picture of the bulk and the surface properties of the catalysts under their working state.

**Fig. 5:**
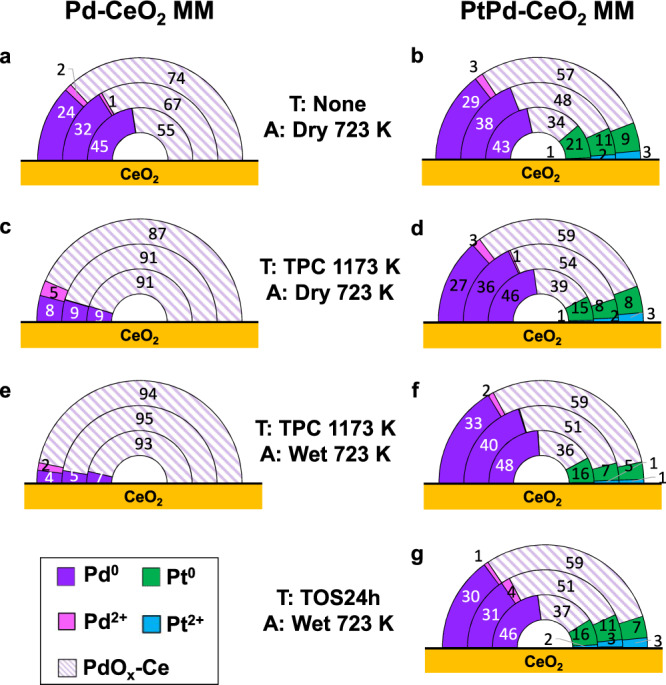
Palladium and Pt atomic fractions and oxidation states calculated for Pd-CeO_2_ MM and PtPd-CeO_2_ MM. **a**–**d** were acquired under dry lean methane oxidation conditions methane combustion mixture and **e**–**g** under wet conditions. The outer semicircle corresponds to the data obtained with KE of 230 eV, the intermediate semicircle corresponds to KE of 450 eV, and the inner semicircle corresponds to KE of 1000 eV. ***T*** indicates the previous pretreatment performed on the catalyst (TPC temperature-programmed combustion, TOS time-on-stream). ***A*** indicates the gaseous atmosphere present during the measurements. The numbers indicate the atomic concentration. MM mechanical milling.

Palladium and Pt atomic fractions and oxidation states obtained with three photon energies are displayed in Fig. 5. Each half pie chart schematically represents an NP, where the three semicircles represent the three sampling depths. The recorded Pd *3d* and Ce *3d* spectra are shown in Figs. S11 and S12, respectively.

Analyses of the Pd *3d* spectra (Fig. S11) reveal the presence of three different oxidation states: (1) metallic Pd at binding energies (BE) between 334.9 and 335.3 eV, (2) a component at BE between Pd^0^ + 0.5 eV and Pd^0^ + 1.2 eV, which corresponds to Pd^2+^, and (3) another oxide component at BE between Pd^0^ + 2.1 eV and Pd^0^ + 2.4 eV. Remarkably, the latter component appears at a BE between those of Pd^2+^ and Pd^4+^. This BE shift could be ascribed to surficial Pd oxide species strongly interacting with ceria^42–44^ and, thus we have named them as PdO_X_-Ce. Under the dry methane oxidation atmosphere at 723 K (Fig. 5a), Pd in the fresh Pd-CeO_2_ MM catalyst is predominantly oxidized, although up to 45% metallic Pd is found at the core of the NPs. Interestingly, a strong core-shell of oxidation states is observed, increasing the amount of oxidized Pd toward the NPs surface. Taking into account the differences between the depths probed with each photon energy, these results indicate that the oxidation phenomenon is strongly located in the first atomic layers. This is also clearly visible in Fig. S11a. Under dry methane oxidation conditions, the Pd relative atomic concentration [Pd/(Pd+Ce)·100] at the surface region is ca. 33 at.% vs. 11 at.% at the subsurface (Table S4), indicating that Pd is finely dispersed over the ceria surface. This fully agrees with the CN extracted from the EXAFS analysis and the HRTEM characterization reported elsewhere^32^, where an amorphous Pd-O-Ce shell ca. 2 nm-thick was identified on the ceria support.

The TPC1173 treatment resulted in strong oxidation of Pd in the three shells, increasing the contribution of the PdO_X_-Ce component to ca. 90% for the three sampling depths, thus losing the core-shell of oxidation states, as seen in Figs. 5c and S11b. A strong restructuring of the surface was also detected as the relative Pd concentration at the surface decreased from about 33 at.% to 10 at.%, while at the subsurface region the Pd relative concentration remained practically constant, suggesting Pd sintering due to the TPC1173 treatment. Upon dosage of steam (wet conditions) at 723 K, the Pd dispersion on the surface did not change (Figs. 5e and S11c and Table S4). This indicates that the TPC1173 aging treatment leads to the reorganization and stabilization of Pd species on the surface notably favoring a more oxidized Pd state under reaction conditions. Remarkably, the oxidation state is preserved after dosing steam and under wet reaction conditions ca. 95 at.% PdO_X_-Ce species are present at the surface.

Interestingly, the monometallic Pt-CeO_2_ MM sample showed a much lower Pt dispersion compared to that of Pd in the monometallic Pd-CeO_2_ MM sample after the TPC1173 (Table S5). The relative Pt atomic concentration is ca. 3 at.% and 5 at.% at the outermost and the subsurface shells, respectively, indicating that a lower Pt dispersion on the CeO_2_ surface is obtained upon milling. This is in agreement with previous HRTEM measurements, where Pt NPs of about 5–10 nm are identified on the ceria support^34^ and in agreement with our SXRD measurements (Table S1). In contrast to the organization of Pd in Pd-CeO_2_ MM, in Pt-CeO_2_ MM Pt shows a core-shell of oxidation states under dry methane combustion conditions at 723 K (Figs. S14 and S15), where 53, 69, and 74 at.% of Pt^2+^ species (with respect to Pt species) are found at the core, subsurface and the outermost layers, respectively, being the rest metallic Pt^0^ (Figs. S14 and S15). Considering that in the results obtained for the core, the outermost layers are also probed, our results indicate that the oxidation is mostly confined to the first atomic layers of the Pt NPs, which are in direct contact with the reaction atmosphere. Under wet methane oxidation conditions at 723 K, the Pt relative atomic concentration remains almost identical as under dry conditions, which as in the case of Pd, indicates that the surface restructuring during the TPC1173 treatment confers stability to the NPs under reaction conditions. The addition of steam did not cause changes to the oxidation state of Pt. These are remarkable findings, since usually Pt is claimed to be in a reduced state under lean methane combustion conditions^23,45^, as our results from in situ bulk SXRD measurements show. Noteworthy, the surface sensitivity provided by AP-XPS reveals that there is Pt^2+^ strongly located at the surface of the NPs, which escapes the detection using bulk-sensitive techniques, coexisting with Pt^0^. In all cases, both under dry and wet lean methane combustion conditions, cerium is mostly present as Ce^4+^ species (>95%) (Table S6).

**Fig. 6:**
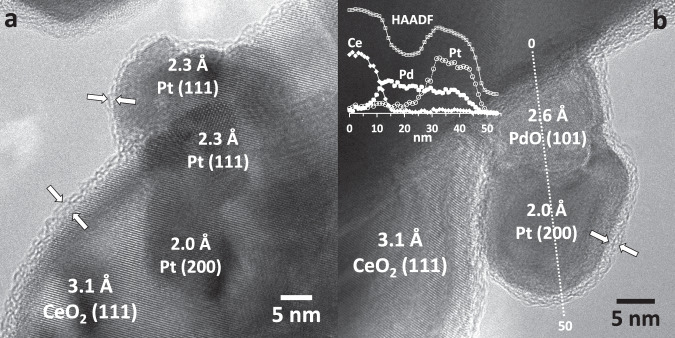
HRTEM images of PtPd-CeO_2_ MM showing the amorphous shell. **a** Fresh PtPd-CeO_2_ MM and **b** after methane combustion under a wet atmosphere at 723 K for 24 h.

The results for the bimetallic PtPd-CeO_2_ MM (Figs. 5 and S11d–f and S13), which is the catalyst with the highest activity and stability, reveal remarkable differences in comparison with the monometallic counterparts. For the fresh PtPd-CeO_2_ MM under the dry lean methane atmosphere at 723 K, the relative total metal atomic concentrations [(Pd + Pt)/(Pd + Pt + Ce)·100] at the surface and the subsurface regions are about 13 and 17 at.%, respectively (Table S7), which in the case of the outermost layer is substantially lower than the relative Pd atomic concentration registered for Pd-CeO_2_ MM (33 at.%). Additionally, the Pd concentration increases toward the NP surface for the bimetallic sample (88 at.% Pd at the surface vs. 78 at.% at the core), leading to a core-shell of compositions with a Pd-rich shell^25^. This is an interesting result, as Pd was milled first with ceria and, then, Pt was added to the milling process. Therefore, this segregation trend indicates that Pt forms larger assemblies while Pd is highly dispersed over the catalyst surface and tends to segregate toward the surface under methane combustion. This is in agreement with the EXAFS results and the HRTEM observations. Figure 6a shows an HRTEM image of the fresh PtPd-CeO_2_ MM catalyst, which shows the coexistence of an amorphous shell of about 2 nm on the ceria support (marked by white arrows) and the Pt NPs (measuring 5–8 nm and identified by lattice fringes at 2.3 and 2.0 Å corresponding to (111) and (200) planes of Pt metal, respectively). Remarkably, the amorphous shell not only covers the ceria support but also the Pt NPs, even though Pt was added in the last step of the milling process. The amorphous shell is identical to that reported previously for monometallic Pd-CeO_2_ MM^32^, and could be the origin of the binding energy shift observed. On the other hand, a core-shell of oxidation states is observed for both Pd and Pt, presenting a larger fraction of oxidized species at the outermost layer. Due to the amorphous nature of this phase, it escapes SXRD detection.

After performing the TPC1173 treatment, a substantially different scenario under the dry mixture at 723 K is observed (Figs. 5d and S11e) compared to the monometallic catalysts. While Pd-CeO_2_ MM exhibited a homogeneous distribution of Pd concentration and oxidation states across the three shells, the bimetallic sample keeps the core-shell of concentrations and oxidation states, as before the TPC1173 treatment. This is additional proof of the role of Pt in the stabilization of Pd. Both Pd and Pt appear more reduced than in their respective monometallic counterparts: under dry reaction conditions at 723 K, 67 at.% Pd is found in PdO_X_-Ce state at the surface, while Pd-CeO_2_ MM is composed of ca. 90 at.% PdO_X_-Ce. On the other hand, Pt appears in a highly reduced state, with 74 at.% and 95 at.% Pt^0^ at the surface and core regions, respectively, in contrast to the previously found values of 26 at.% and 47 at.% for the monometallic Pt-CeO_2_ MM.

Under the wet feed at 723 K, both metals undergo a slight reduction, being the innermost layers of the NPs more reduced, and being Pt almost fully reduced. At the same time, no further restructuring of the metals is observed (Figs. 5f and S11f). This reveals that the interaction between both metals leads to a higher degree of reduction of the first layers of both Pd and Pt and a more stable system than Pd-CeO_2_ MM. To confirm this, the sample treated in the laboratory for 24 h under TOS wet lean methane combustion conditions was studied by AP-XPS directly under wet conditions at 723 K. As seen in Fig. 5g, similar results are obtained, thus demonstrating the robustness of the PtPd-CeO_2_ MM system. Figure 6b corresponds to a representative HRTEM image of the bimetallic sample after the TPC1173 treatment and 24 h under TOS wet. A surprising reorganization was found after the TPC cycle, as reported elsewhere^34^: under lean methane combustion unique structures with a PdO foot anchored to the ceria support and covered by a Pt head grew throughout the sample as seen in Fig. S16. The mushroom-like structures remain during the wet operation. Therefore, Pt NPs are in close contact with PdO, which in turn exhibits a strong contact with the ceria support. These assemblies are embedded in an amorphous shell, similar to that recognized in the fresh sample (Fig. 6a). Additionally, the energy dispersive X-ray spectroscopy profile analysis carried out along the Ce-PdO-Pt assembly shown in the inset of Fig. 6b clearly reveals the presence of Pd in the Pt NP, as in the line scan performed both Pd and Pt signals are detected between 30 and 45 nm. These observations match well the in situ SXRD and XAS results as well as the AP-XPS. Notably, the Pd-rich amorphous shell, which is thought to be the origin of the PdO_X_-Ce species, is preserved during methane oxidation at 723 K, even under wet conditions. The intimate contact of the PdO_X_-Ce species with the Pt-Pd/PdO assemblies constitutes an unprecedented architecture, which can be ascribed to the origin of the catalytic activity and robustness of the bimetallic system. This nanostructure is only attained by mechanochemical synthesis.

Studying the surface and subsurface regions by AP-XPS using three photon energies and combining these results with in situ SXRD and XAS, and HRTEM observations shed light on the evolution of the surface and the bulk structure of ceria-supported Pd, Pt, and bimetallic Pd-Pt catalysts prepared by mechanochemical methods and by conventional incipient wetness impregnation during the oxidation of methane under dry and wet conditions, relevant for natural gas-fueled vehicles. We have demonstrated that both the composition and the synthesis method have a direct and strong impact on the arrangement and chemical environment of Pd and Pt as well as on their evolution under reaction conditions. In the milled catalysts, oxidized PdO_X_-Ce species strongly interacting with ceria are preserved during methane oxidation under wet conditions, which are likely related to the unique Pd-rich amorphous shell originated from the mechanochemical preparation method, and stabilized by intimate contact with Pt-Pd/PdO assemblies originated under TPC reaction conditions. Our data reveal that the structure of mechanochemically-prepared catalysts is more dynamic, enabling the progressive formation of PdO at the expense of metallic Pd even during wet methane combustion, due to the intimate interaction obtained between Pd and CeO_2_ in the milled catalysts, which modulates the PdO/Pd ratio and reaches a value close to unity under wet reaction conditions. This transformation parallels the increase in methane conversion observed, therefore avoiding the well-known deactivation of Pd-based catalysts under wet conditions. These data demonstrate that unique structural properties are achieved during the mechanochemical synthesis, an eco-friendly and readily scalable synthesis method, which leads to superior methane conversion even under the presence of steam.

## Methods

### Catalysts synthesis

The CeO_2_ support was synthesized by precipitation using H_2_O_2_^46^. Cerium nitrate (Ce(NO_3_)_2_·6H_2_O, Treibacher Industrie AG) was dissolved in deionized water (0.2 M) and kept under stirring before an appropriate amount of hydrogen peroxide (H_2_O_2_, Aldrich, 35%) was poured into the solution to obtain a molar H_2_O_2_:Ce ratio of 3. The precipitation of the precursor was then obtained by addition of aqueous ammonium hydroxide (NH_4_OH, Aldrich, 30%) to reach a pH of 10.5. The slurry was stirred for 4 h, washed with deionized water, and filtered. The precipitate was then dried in static air at 393 K for 15 h and subsequently calcined in static air at 1173 K for 3 h. The surface area after calcination was 2.6 m^2^/g for ceria.

Palladium and platinum were incorporated to the ceria support by milling Pd nanoparticles (NPs) (Aldrich, surface area 40 m^2^/g, mean particle size 10 μm) or Pt NPs (Sigma-Aldrich, surface area 33 m^2^/g, mean particle size ≤20 μm) with ceria in a Pulverisette 23 Mini-Mill for 10 min at a frequency of 15 Hz, using a 15 ml zirconia bowl with 1 grinding ball made of ZrO_2_ (diameter = 15 mm, weight = 10 g, ball-to-powder ratio = 10). The required amount of Pd or Pt was added to obtain a nominal loading of each metal of 1.5 wt%. For the bimetallic catalysts, the previous procedure was followed to incorporate one metal and subsequently, the second metal was added. The catalysts have been named PtPd-CeO_2_ MM when Pd was first milled with ceria and Pt was added subsequently, and PdPt-CeO_2_ MM, when the addition order was inverted and Pt was first milled and then Pd was added. The catalysts were investigated without further treatments. Therefore, in the catalysts prepared by mechanochemical methods, no solvents are used.

For the preparation of IWI catalysts, CeO_2_ was impregnated with an appropriate amount of an aqueous solution of palladium nitrate (Pd(NO_3_)_2_, 4.8 wt% Pd, 99.999%, Sigma-Aldrich) and tetraammineplatinum (II) nitrate ([Pt(NH_3_)_4_](NO_3_)_2_, 99%, Strem Chemicals) to reach a nominal loading of 1.5 wt% Pd and 1.5 wt% Pt. The resulting catalyst was dried overnight at 373 K and then calcined in static air for 3 h at 1173 K.

### Catalytic methane oxidation measurements

The catalytic activity evaluation was carried out in a quartz tubular reactor, loaded with 120 mg of catalyst powder supported on a quartz wool bed. The total flow rate was set at 180 ml/min, corresponding to a GHSV of about 180,000 h^−1^. The gas inlet composition was 0.5 vol% CH_4_ and 2 vol% O_2_ in He for dry experiments. For the wet atmosphere experiments, 10 vol% H_2_O was added by using a high-performance liquid chromatography (HPLC) pump that provided a flow of deionized water, which was then evaporated by heating tapes to obtain the additional 10 vol% of steam in the feed gas. A K-type thermocouple was placed inside the reactor close to the catalyst bed for continuous monitoring of the sample temperature. The reactor was placed inside a furnace equipped with a PID controller.

### In situ synchrotron X-ray powder diffraction (SXRD) measurements

The in situ synchrotron XRD measurements were carried out at the Materials Science and Powder Diffraction beamline (BL) of the ALBA synchrotron (Cerdanyola del Vallès, Barcelona, Spain). The BL was set at an energy corresponding to *λ* = 0.5157 Å wavelength and all data were collected in transmission mode. The catalysts were loaded in quartz capillaries of internal diameter 0.58 ± 0.1 mm (Hilgenberg GmbH) and immobilized with quartz wool. The gases (methane, oxygen, argon) were dosed using a gas delivery system available at the BL (the gas control system from ITQ-ALBA) using independent mass flow controllers. The gas inlet composition was 0.5 vol% CH_4_ and 2 vol% O_2_ in Ar for dry experiments. For the wet atmosphere experiments, water was dosed flowing the reactants mixture through a saturator filled with milli-Q water kept at RT. The sample temperature was controlled using a calibrated hot blower. A mass spectrometer was connected at the reactor outlet to monitor the gaseous effluent of the reactor. The diffractograms were recorded between 5 and 46.8°, with a 0.006° step.

The Rietveld refinements were performed using the GSAS-II software^47^. The refinements were performed including as reference standard materials CeO_2_ (01-080-5549), Pd (00-046-1043), Pt (00-004-0802), and PdO (00-041-1107). A model Pd_0.5_Pt_0.5_ (01-072-2839) alloy was also considered in the refinements.

The crystallite size was determined using the Scherrer Eq. (1):

1 $$\tau=\frac{K\lambda }{\beta {\cos }\theta }$$

where *τ* is the mean crystallite size; *K* is a shape factor (*K* = 0.9 was chosen in this work), *λ* = 0.5157 Å, and *β* is the full width at half maximum. Table S1 lists the results obtained for the investigated catalysts.

### In situ X-ray absorption fine structure spectroscopy (XAS) measurements

The in situ XAS measurements were performed at the Core Level Absorption & Emission Spectroscopies BL of the ALBA synchrotron. The catalysts were pelletized and diluted with boron nitride to optimize the sample absorption. The samples were mounted in the solid-gas reactor cell existing at the beamline^48^. This cell allows controlling the gas dosage using the same gas control system defined for the in situ XRD measurements (the ITQ-ALBA gas control system) and to control the sample temperature. A maximum temperature of ~1000 K (depending on the gaseous mixture) can be reached with this cell. Pd K-edge spectra were recorded in transmission mode. A Pd reference foil was also measured together with the spectra of the samples for energy calibration. A Si(311) monochromator was used for energy selection. The appropriate mixture of inert gases (He, N_2_, Ar, Kr, and Xe) was selected to fill the ionization chambers that are used as X-ray detectors in the transmission experiments. Pt L_3_-edge spectra were recorded in fluorescence mode using a 6-element silicon drift detector.

The in situ measurements consisted of dosing the lean methane combustion mixture (0.5 CH_4_ + 2 O_2_ + 97.5 He—dry mixture) at RT, increasing the temperature using a 10 Kmin^–1^ ramp. The catalysts were measured as pressed pellets optimizing the weight of catalyst for the Pd K-edge and the Pt L_3_-edge. The reactants flows were adjusted to keep the same weight-to-flow ratio as the ratio used in the catalytic tests carried out in the laboratory. X-ray absorption near-edge spectra (XANES) were acquired continuously when the temperature was changed and extended X-ray absorption fine structure spectra (EXAFS) were acquired under steady temperatures and at RT once the in situ series was finished. At least, three scans were acquired at each temperature step to ensure spectral reproducibility and a good signal-to-noise ratio. Data analysis and treatment has been performed using the Athena software^49^. EXAFS data analysis has been performed using the Arthemis software^49^, and phase and amplitudes have been calculated using the FEFF6 code.

Since the maximum temperature reachable at the BL was 1000 K, the maximum temperature reached during the experiments under the dry mixture was 923 K. Therefore, to study the catalysts under wet lean methane combustion conditions, the TPC pretreatment at 1173 K under 0.5 CH_4_ + 2 O_2_ + 97.5 He was performed in our laboratories under the same dry mixture. The protocol used for the wet measurements was as follows: the pretreated catalysts were initially exposed to the dry reaction mixture up to 723 K to refresh the catalysts (recording spectra), the temperature was lowered to 443 K, at which water was introduced to the reactants feed and then the temperature was increased step-wise up to 723 K, where 2 h measurements were carried out (see Fig. S1). For the wet measurements, 10 vol.% water was added using an HPLC pump (Knauer Smartline). The reaction series is schematized in Fig. S1.

We fitted the EXAFS data to obtain quantitative information. We started fitting the first shell of a Pd foil to obtain the value of the amplitude reduction factor, S_0_^2^. The value obtained was used for the subsequent analyses of the EXAFS data of our catalysts. The fits of the reference foil and the catalysts was performed in the same ranges in k- and R-space. Only up to the first Pd-M coordination shell was fitted.

### Synchrotron near-ambient pressure X-ray photoelectron spectroscopy (AP-XPS) measurements

The AP-XPS measurements were carried out at the NAPP end station from the CIRCE beamline of the ALBA Synchrotron Light Source. A commercial PHOIBOS 150 NAP energy analyzer (SPECS GmbH) equipped with four differentially pumped stages connected by small apertures was used to analyze the emitted photoelectrons. The beam spot size at the sample was ~100 × 300 μm^2^ (horizontal × vertical). The spectra were acquired with 20 eV pass energy and 0.05 eV energy step. The sample pressure was kept at 1 mbar for all measurements regulating the active pumping. For that, the gases composing the reactants mixture were dosed to the analysis chamber using independent mass flow controllers and regulating and stabilizing the pressure inside the analysis chamber at 1 mbar for all experiments with a system of vacuum valves. A constant flow of 25 ml/min for the dry mixture (1 CH_4_ + 4 O_2_ + 20 N_2_) and of 27.5 ml/min for the wet mixture (1 CH_4_ + 4 O_2_ + 20 N_2_ + 2.5 H_2_O) was introduced into the analysis chamber. During these measurements, the maximum reachable temperature was ca. 973 K and, therefore, the same protocol followed for the in situ XAS measurements were used for the AP-XPS studies to make sure that under the dry reaction mixture 1173 K were reached. Figure S1 schematically represents the protocol followed. Therefore, to carry out the wet measurements, samples pretreated under TPC1173 in the laboratory were studied. Then the dry mixture was dosed to the analysis chamber and the temperature was increased to 723 K. After that, the samples were cooled down to 443 K under the dry reaction mixture and at this temperature, steam was introduced in the analysis chamber. Then the samples under wet conditions were studied up to 723 K (see Fig. S1b).

The sample temperature was controlled using an infrared laser (*λ* = 808 nm) focused on a W plate on top of which the samples were mounted. The temperature was monitored during all the experiments with a K-type thermocouple in direct contact with the samples.

For each reaction condition, XP spectra of Ce *3d*, Pd *3d*, Pt *4f*, O *1s*, and C *1s* regions were studied. To obtain depth profile information and be able to determine the metal concentration and oxidation state at different depths, each spectral region was excited with three different photon energies, keeping the kinetic energy (KE) of the generated Pd *3d*, Pt *4f*, Ce *3d*, C *1s*, and O *1s* photoelectrons constant at ~1000, 450, and 230 eV. The corresponding inelastic mean free paths (IMFP) for each KE calculated for the corresponding spectral regions taking into account pure metals are listed in Table S3^50^. Therefore, to calculate approximately the IMFP in our measurements, a Pd:Pt = 1:1 composition was assumed for the NPs, which allows us to calculate the IMFP of the photoelectrons in our NPs as the mean value of the IMFP for the two pure different elements.

The atomic fractions of Pd and Pt were obtained from the calibrated Pd *3d* and Pt *4f* peak areas. The relative sensitivity factors (RSF) were calculated taking into account the ionization cross-section, the photon flux corresponding to the photon energy used for each measurement^51^, and the transmission function of the analyzer. The AP-XPS spectra were analyzed without energy calibration. The presence of the gases and temperature led to minor shifts due to charge. No corrections for angular distribution were applied since the angle between the analyzer axis and the incoming synchrotron radiation horizontal linear polarization vector is 54.7°, the magic angle^52^. As the KE was kept constant, the mean free path parameter was set to 1 in the RSF. The atomic concentration of each metal was calculated taking into account the corrected areas and using the formula: (M + N)/(M + N + Ce)·100; M, N = Pd or Pt.

## Supplementary information

Supplementary Information

## Data Availability

The data that support the findings of this study are included in the published article (and its Supplementary Information) or available from the corresponding authors on reasonable request.
