# Supplementary material for: Investigation of the evolution of Pd-Pt supported on ceria for dry and wet methane oxidation
Source: Nat Commun. 2022 Aug 29;13:5080. doi: 10.1038/s41467-022-32765-4 (PMC9424231; doi:10.1038/s41467-022-32765-4)
Supplement: Supplementary file 1 — Supplementary Information [file 41467_2022_32765_MOESM1_ESM.pdf]

Supplementary Information

Investigation of the evolution of Pd-Pt supported on ceria for dry and wet methane oxidation

Núria. J. Divins[1,2,3], Andrea Braga[1,2], Xavier Vendrell[1,2], Isabel Serrano[1], Xènia Garcia[1,2,3], Lluís Soler[1,2,3], Ilaria Lucentini[1,2], Maila Danielis[4], Andrea Mussio[4], Sara Colussi[4], Ignacio J. Villar-Garcia[5], Carlos Escudero[5], Alessandro Trovarelli[4], Jordi Llorca[1,2,3*]*

[1]Institute of Energy Technologies, Universitat Politècnica de Catalunya, EEBE, Eduard Maristany 10-14, 08019 Barcelona, Spain

[2]Department of Chemical Engineering, Universitat Politècnica de Catalunya, EEBE, Eduard Maristany 10-14, 08019 Barcelona, Spain

[3]Barcelona Research Center in Multiscale Science and Engineering, Universitat Politècnica de Catalunya, EEBE, Eduard Maristany 10-14, 08019 Barcelona, Spain

[4]Dipartimento Politecnico, Università di Udine, and INSTM, via del Cotonificio 108, 33100 Udine, Italy

[5]ALBA Synchrotron Light Source, Carrer de la Llum 2-26, 08290 Cerdanyola del Vallès, Barcelona, Spain

Corresponding Authors:

Dr. Núria J. Divins (nuria.jimenez.divins@upc.edu)

Prof. Dr. Jordi Llorca (jordi.llorca@upc.edu)

Supplementary Discussion

EXAFS analyses were performed for Pd-CeO_2 MM (MM: mechanical milling) and PtPd-CeO_2 MM catalysts. A good fit can be obtained by only considering a Pd-M contribution (M=Pd, Pt). Nevertheless, the fits for both samples improve when a Pd-O contribution is added, compatible with the presence of Pd-O bonds (Figure S10 shows the fit results for both samples). The *R*-space range chosen to fit the EXAFS spectra $\chi(k)k^2$ was from $R_{\min}$ = 1.3 Å up to $R_{\max}$ = 3.3 Å. The Fourier transform was carried out in the *k* range from 2 Å^{-1} up to 11.6 Å^{-1}. For the first Pd-M and Pd-O shells, coordination numbers *N*, interatomic distances *R*, corrections to photoelectron reference energies ΔE_0, and Debye-Waller disorder factor σ^2_{Pd-Pd} and σ^2_{Pd-O}were fitted. Figure S10 shows the results of the fits and in Table S2 the results are listed.

The EXAFS data acquired at RT for the TPC1173 samples (Fig. 4) indicate that during the TPC1173, Pd strongly oxidized and transformed to bulk PdO. On the other hand, both bimetallic catalysts (MM and IWI- IWI: incipient wetness impregnation-) present Pd-O bonds at ca. 1.5 Å, but with a lower contribution than for the monometallic catalyst. In both bimetallic catalysts, a clear Pd-M (M=Pd or Pt) contribution is observed, which is not visible in the monometallic catalyst, indicating that in both bimetallic catalysts Pd is present in a mixture of oxidation states.

Supplementary Tables

Table S1. Particle size determined from the Scherrer equation for the indicated catalysts. The calculations have been done with the peak corresponding to Pd(111) or Pt(111).

Sample - Treatment	Particle size (nm)
Pd-CeO_2 MM as-prepared	12.5
Pt-CeO_2 MM as-prepared	10.9
PtPd-CeO_2 MM as-prepared	9.5
PtPd/CeO_2 IWI as-prepared (after calcination at 1173 K)	42.7
Pd-CeO_2 MM TPC1173 (PdO)*	32.9
Pt-CeO_2 MM TPC1173	66.6
PtPd-CeO_2 MM TPC1173	26.1
PtPd/CeO_2 IWI TPC1173	94.3

***** The reported value has been calculated with the peak corresponding to PdO (110). TPC: Temperature-programmed combustion. MM: mechanical milling. IWI: incipient wetness impregnation.

Table S2. Best-fit parameters extracted from the analysis of Pd K-edge EXAFS spectra for the as-prepared Pd-CeO_2 MM and PdPt-CeO_2 MM catalysts. Included are the coordination numbers (CN), the bond lengths (R), Debye-Waller factors (σ^2) for Pd—O and Pd—M bonds, and energy shift (ΔE0). The values in parenthesis are the standard errors in the last digit. The amplitude reduction factor obtained from the fit of the Pd foil is S_0^2=0.84.

Sample	CN_{Pd-Pd}	R_{Pd-Pd} (Å)	σ^2_{Pd-Pd} (Å^2)	ΔE_0 (eV)	N_{Pd-O}	R_{Pd-O} (Å)	σ^2_{Pd-O} (Å^2)	ΔE_0 (eV)	R factor
Pd-CeO_2 MM – as prepared	7.5(6)	2.734(4)	0.006(1)	-5.9(4)	0.7(2)	2.05(4)	0.002(2)	7(3)	0.007
PdPt-CeO_2 MM – as prepared	6.9(4)	2.733(3)	0.006(1)	-6.2(4)	1.0(2)	2.04(2)	0.002(1)	5(2)	0.004

MM: mechanical milling.

Table S3. Calculated IMFP for the Pd *3d*, Pt *4f* and Ce *3d* spectral regions corresponding to the listed kinetic energies.

	IMFP (nm)		
**Region**	**230 eV**	**450 eV**	**1000 eV**
**Pd *3d***	0.7	1.0	1.8
**Pt *4f***	0.5	0.8	1.3
**Ce *3d***	0.7	1.1	2.0

Table S4. AP-XPS results for Pd-CeO_2 MM catalyst, indicating the pretreatment carried out on the catalyst and the gaseous atmosphere dosed during the measurement. The Pd atomic percentage found for the different regions is listed.

Treatment – Atmosphere	KE (eV)	Region	At.% Pd
**None** **Dry 723 K**	230	Surface	33.0
	450	Subsurface	11.4
	1000	Core	-
**TPC1173 K** **Dry 723 K**	230	Surface	9.6
	450	Subsurface	14.5
	1000	Core	-
**TPC1173 K** **Wet 723 K**	230	Surface	10.0
	450	Subsurface	14.2
	1000	Core	-

*Dry mixture: 1 $mLmin^{-1}$ CH_4, 4 $mLmin^{-1}$ O_2 and 20 $mLmin^{-1}$ N_2. 25 $mLmin^{-1}$ total flow.

*Wet mixture: 1 $mLmin^{-1}$ CH_4, 4 $mLmin^{-1}$ O_2, 2.5 $mLmin^{-1}$ H_2O and 20 $mLmin^{-1}$ N_2. 27.5 $mLmin^{-1}$ total flow.

TPC: Temperature-programmed combustion. MM: mechanical milling.

Table S5. AP-XPS results for Pt-CeO_2 MM catalyst, indicating the pretreatment carried out on the catalyst and the gaseous atmosphere dosed during the measurement. The Pt atomic percentage found for the different regions is listed.

Treatment – Atmosphere	KE (eV)	Region	At.% Pt
**TPC1173 K** **Dry 723 K**	230	Surface	3.0
	450	Subsurface	4.9
	1000	Core	-
**TPC1173 K** **Wet 723 K**	230	Surface	2.7
	450	Subsurface	5.0
	1000	Core	-

TPC: Temperature-programmed combustion. MM: mechanical milling. IWI: incipient wetness impregnation.

Table S6. AP-XPS results for **a;** Pd-CeO_2 MM and **b;** PtPd-CeO_2 MM catalysts, indicating the pretreatment carried out on the catalyst and the gaseous atmosphere dosed during the measurement. The percentage of Ce^{4+} species is listed.

a. Pd-CeO_2 MM catalyst

**Treatment – Atmosphere**	**KE (eV)**	**Region**	**At.% Ce^{4+}** [Ce^{4+}/(Ce^{4+}+ Ce^{3+})·100]
**None Dry 723 K**	230	Surface	93.6
	450	Subsurface	95.0
	1000	Core	-
**TPC1173 K Dry 723 K**	230	Surface	96.7
	450	Subsurface	95.9
	1000	Core	-
**TPC1173 K Wet 723 K**	230	Surface	96.0
	450	Subsurface	94.7
	1000	Core	-

TPC: Temperature-programmed combustion. MM: mechanical milling. IWI: incipient wetness impregnation.

b. PtPd-CeO_2 MM catalyst

**Treatment – Atmosphere**	**KE (eV)**	**Region**	**At.% Ce^{4+}** [Ce^{4+}/(Ce^{4+}+ Ce^{3+})·100]
**None Dry 723 K**	230	Surface	97.8
	450	Subsurface	97.8
	1000	Core	
**TPC1173 K Dry 723 K**	230	Surface	98.0
	450	Subsurface	97.8
	1000	Core	-
**TPC1173 K Wet 723 K**	230	Surface	98.3
	450	Subsurface	97.9
	1000	Core	-

TPC: Temperature-programmed combustion. MM: mechanical milling. IWI: incipient wetness impregnation.

Table S7. AP-XPS results for PtPd-CeO_2 MM catalyst, indicating the pretreatment carried out on the catalyst and the gaseous atmosphere dosed during the measurement.

**Treatment – Atmosphere**	**KE (eV)**	**At. % metals** [(Pd+Pt)/(Pd+Pt+Ce)·100]	**Pd/Ce**	**Pt/Ce**	**At. % Pd** [Pd/(Pd+Pt)·100]	**At. % Pt** [Pt/(Pd+Pt)·100]
**None Dry 723 K**	230	12.5	0.13	0.02	88.4	11.6
	450	17.1	0.18	0.03	86.2	13.8
	1000	-	-	-	77.9	22.1
**TPC1173 K Dry 723 K**	230	10.9	0.11	0.01	89.1	10.9
	450	12.9	0.13	0.02	89.8	10.2
	1000	-	-	-	84.2	15.8
**TPC1173 K Wet 723 K**	230	8.2	0.08	0.01	93.1	6.9
	450	12.5	0.13	0.01	91.9	8.1
	1000	-	-	-	84.3	15.7

TPC: Temperature-programmed combustion. MM: mechanical milling. IWI: incipient wetness impregnation.

Supplementary Figures

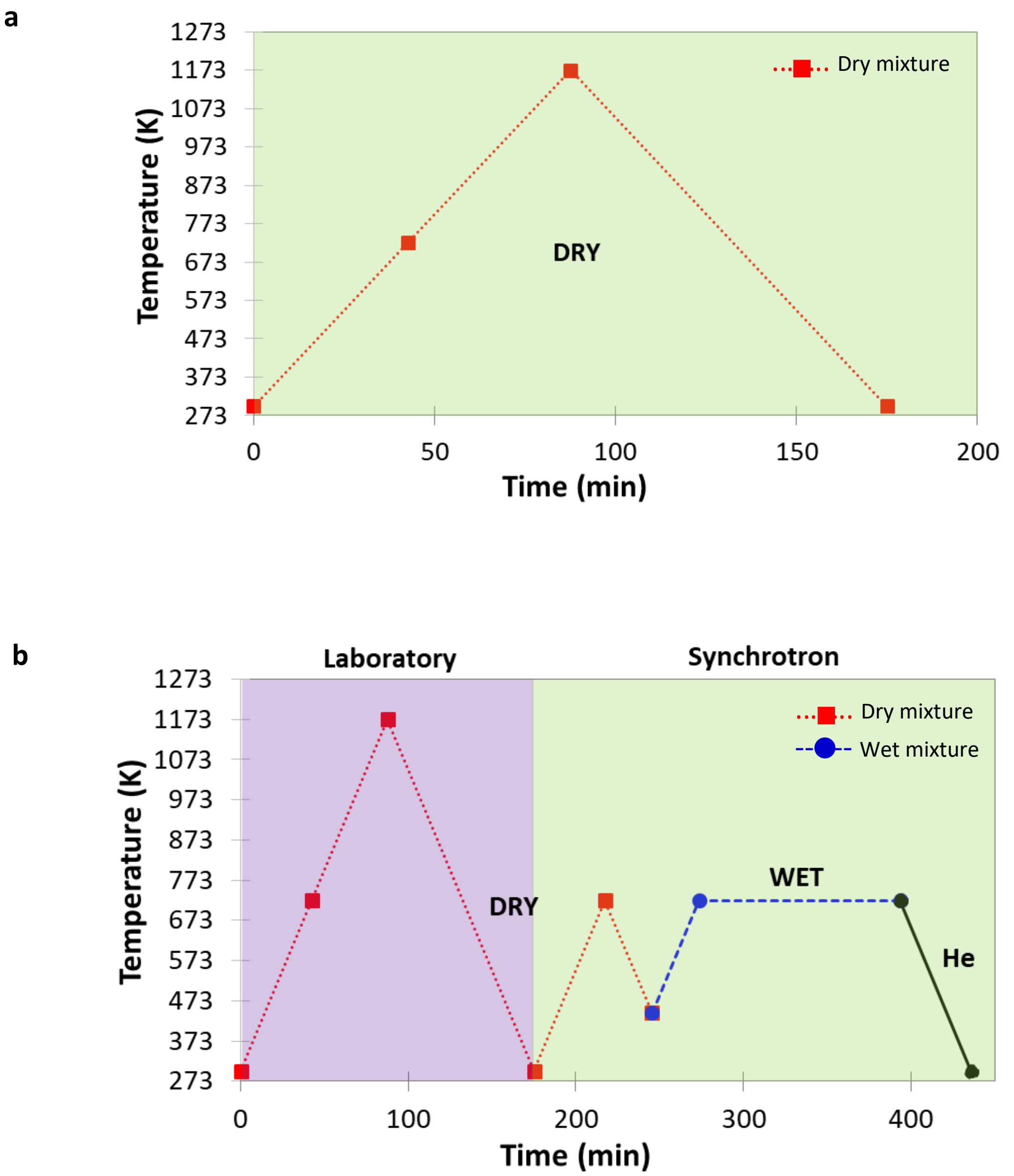

Figure S1. Schematic representation of the reaction series for samples fresh and pretreated samples. a, fresh; **b,** pretreated samples in our laboratories under TPC1173. In **b**, the purple region depicts the part of the measurement carried out in our laboratory and in green at the synchrotron. The squares correspond to the treatment performed under dry lean methane combustion mixture and the circles represent the wet reaction (0.5 CH_4 + 2 O_2 + 97.5 He +10 H_2O). TPC: Temperature-programmed combustion.

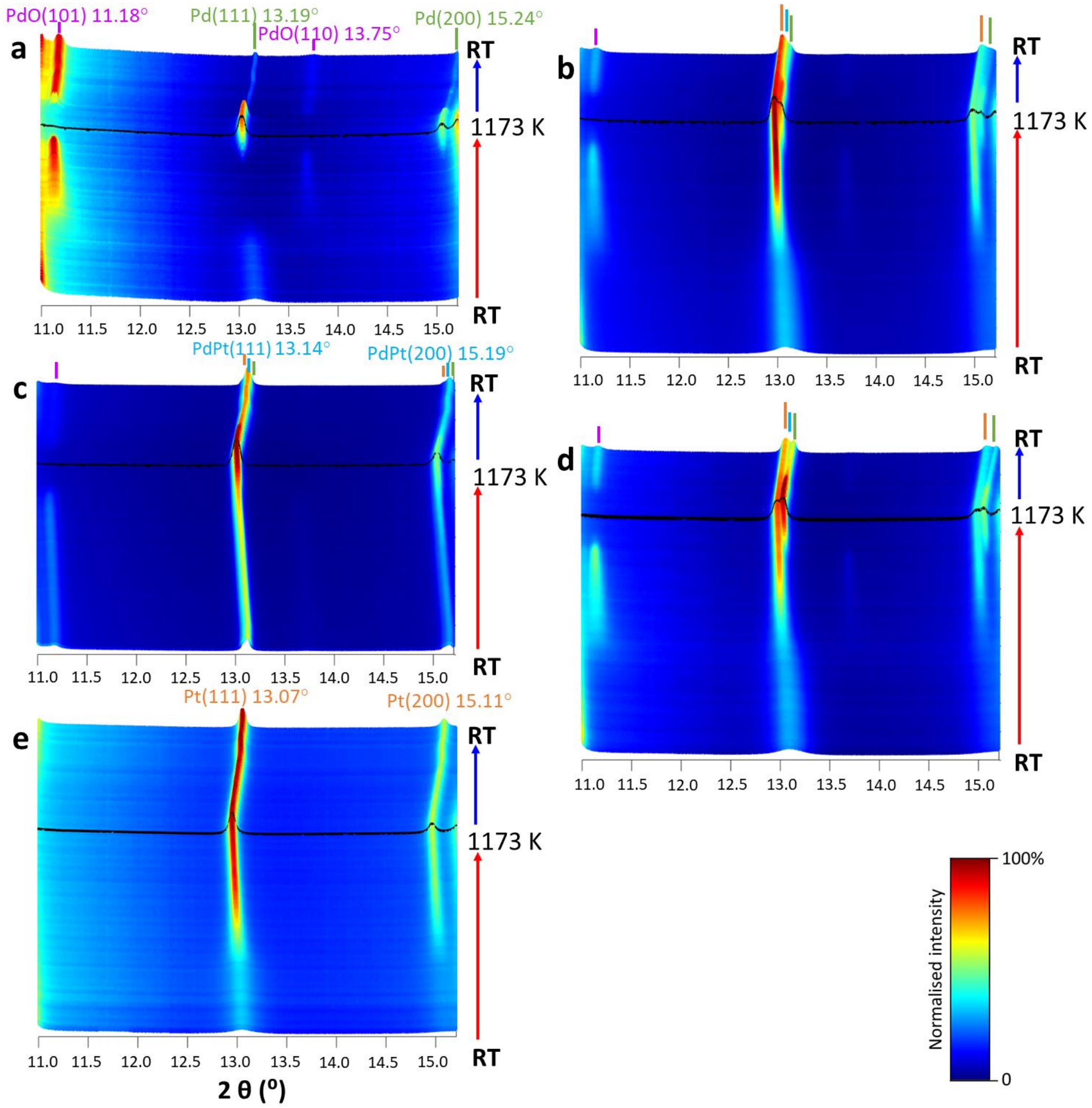

Figure S2. Series of diffractograms of the investigated catalysts acquired during dry lean methane combustion. The diffractograms were acquired ramping up from room temperature (RT) to 1173 K (marked with a red arrow) and cooling down (marked with a blue arrow), corresponding to a TPC1173 treatment for samples: **a**; Pd-CeO_2 MM; **b**; PtPd-CeO_2 MM; **c**; PdPt/CeO_2 IWI; **d**; PdPt-CeO_2 MM; **e**; Pt-CeO_2 MM. The intensity of the diffractograms was normalized in the 2θ range 11.0-15.2°. TPC: Temperature-programmed combustion. MM: mechanical milling. IWI: incipient wetness impregnation.

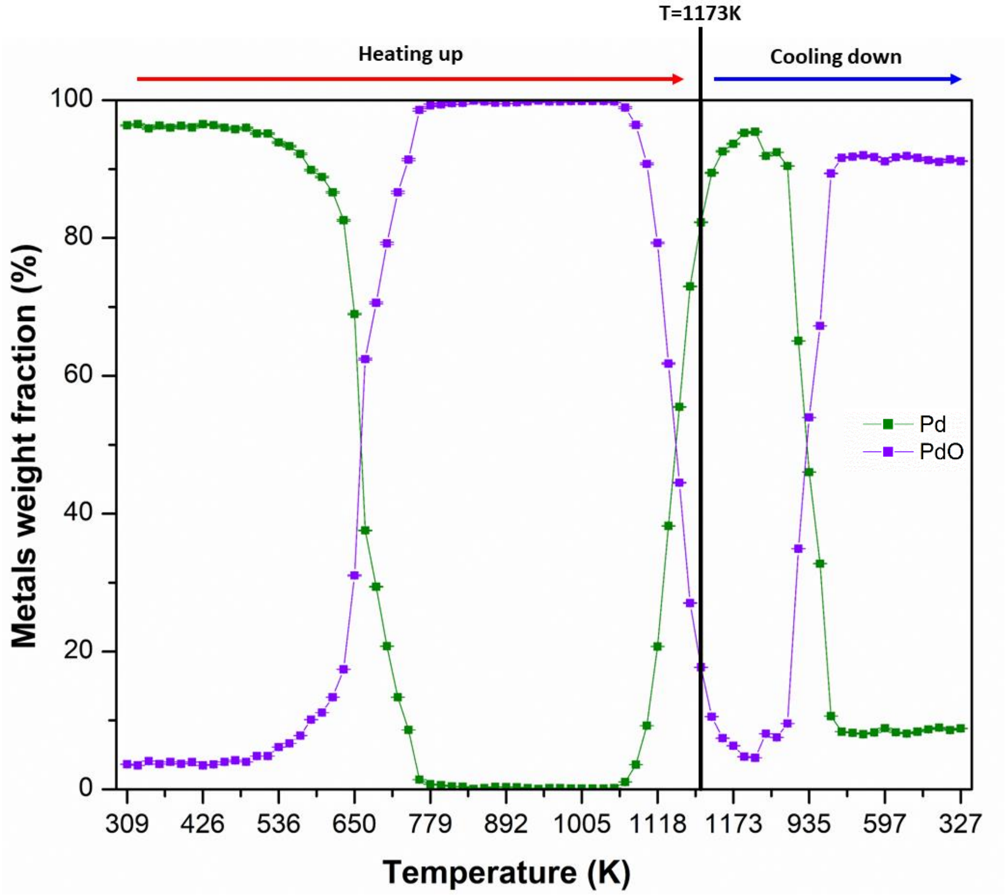

Figure S3. **Evolution of the normalized weight fraction of the identified phases as a function of temperature for Pd-CeO_2 MM.** The weight fractions were obtained from the Rietveld refinement of the diffractograms acquired during dry lean methane oxidation. (TPC1173 treatment). TPC: Temperature-programmed combustion. MM: mechanical milling.

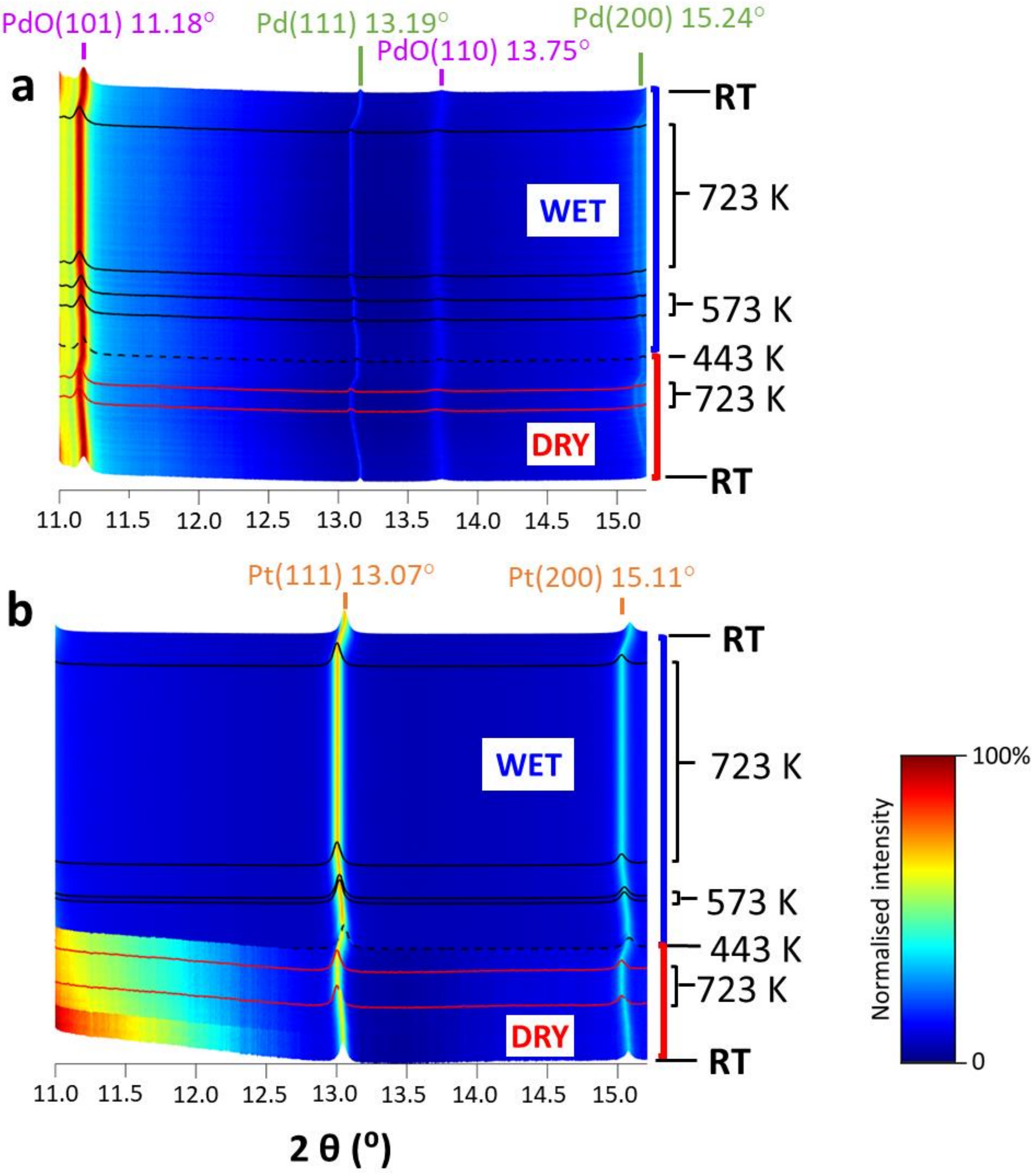

Figure S4. Series of diffractograms of the investigated catalysts acquired during wet lean methane combustion. The diffractograms were acquired following the protocol shown in Figure S1b for samples already exposed to a TPC1173 treatment: **a**; Pd-CeO_2 MM; **b**; Pt-CeO_2 MM. The intensity of the diffractograms was normalized in the 2θ range 11.0-15.2°. TPC: Temperature-programmed combustion. MM: mechanical milling.

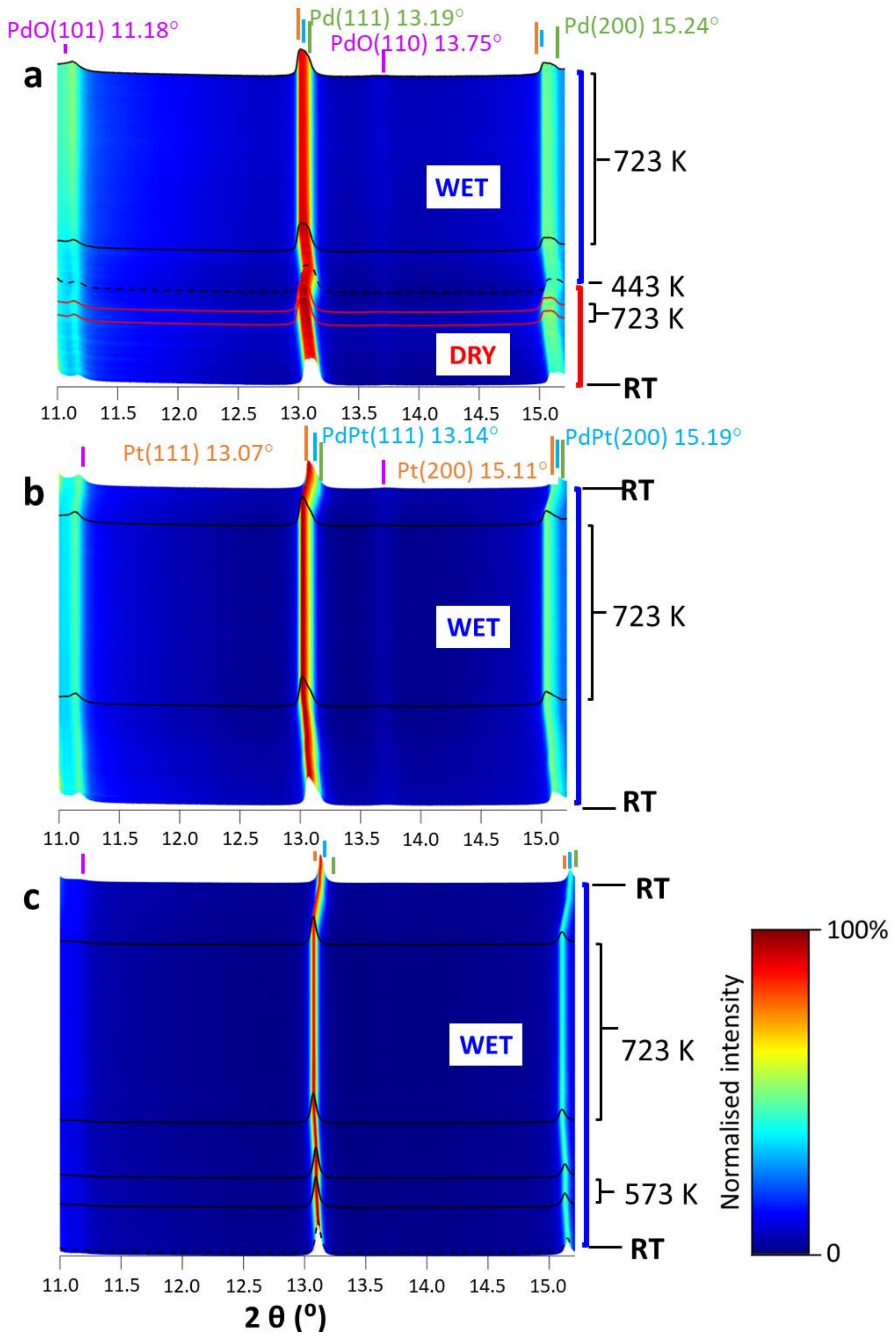

Figure S5. Series of diffractograms of the investigated catalysts acquired during wet lean methane combustion. The diffractograms were acquired following the protocol shown in Figure S1b for samples already exposed to a TPC1173 treatment: **a**; PtPd-CeO_2 MM; **b**; PtPd-CeO_2 MM TOS WET 24h; **c**; PdPt/CeO_2 IWI. The intensity of the diffractograms was normalized in the 2θ range 11.0-15.2°. TPC: Temperature-programmed combustion. TOS: Time-on stream. MM: mechanical milling. IWI: incipient wetness impregnation.

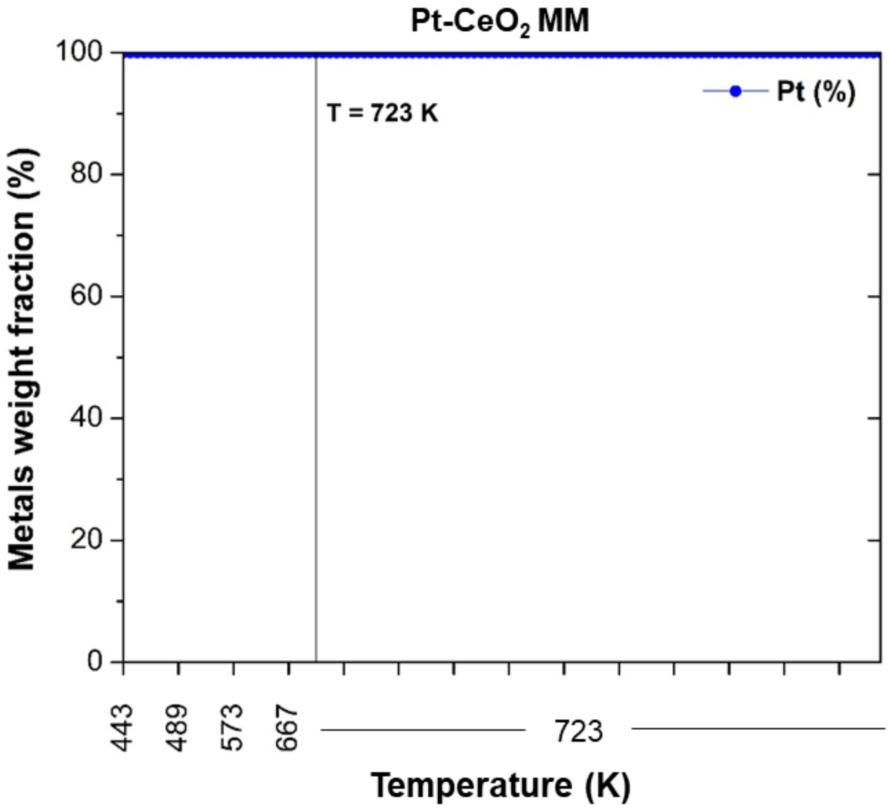

Figure S6. Evolution of the normalized weight fraction of the identified phases as a function of temperature for Pt-CeO$_2$ MM. The weight fractions were obtained from the Rietveld refinement of the diffractograms acquired during wet lean methane oxidation at different temperatures for Pt-CeO$_2$ MM. MM: mechanical milling.

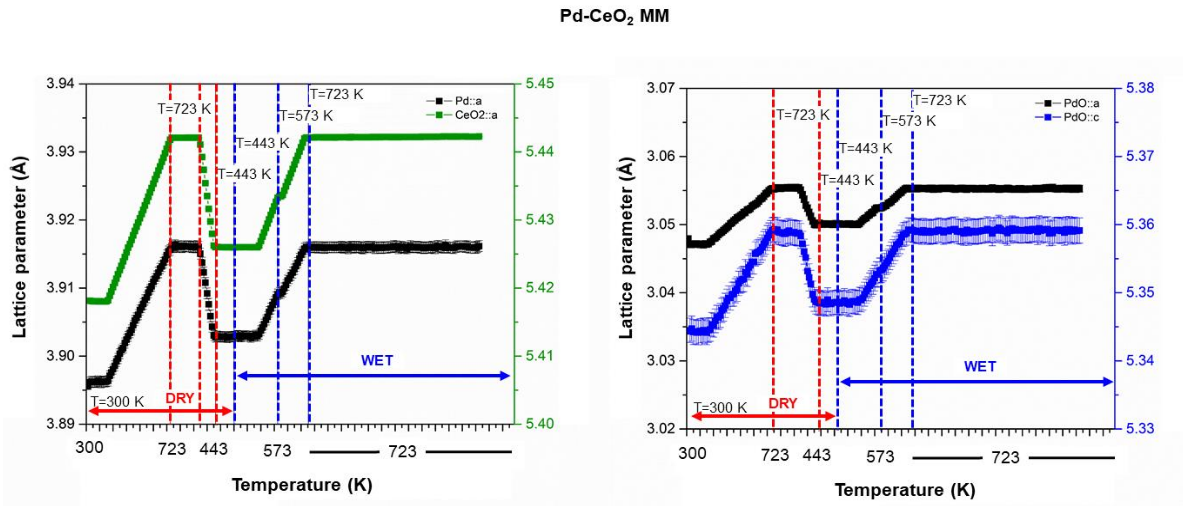

Figure S7. Lattice parameter obtained from the sequential Rietveld refinement of the diffractograms acquired during wet methane combustion for the monometallic Pd-CeO_2 MM catalyst. The error bars correspond to the standard deviation. MM: Mechanical milling.

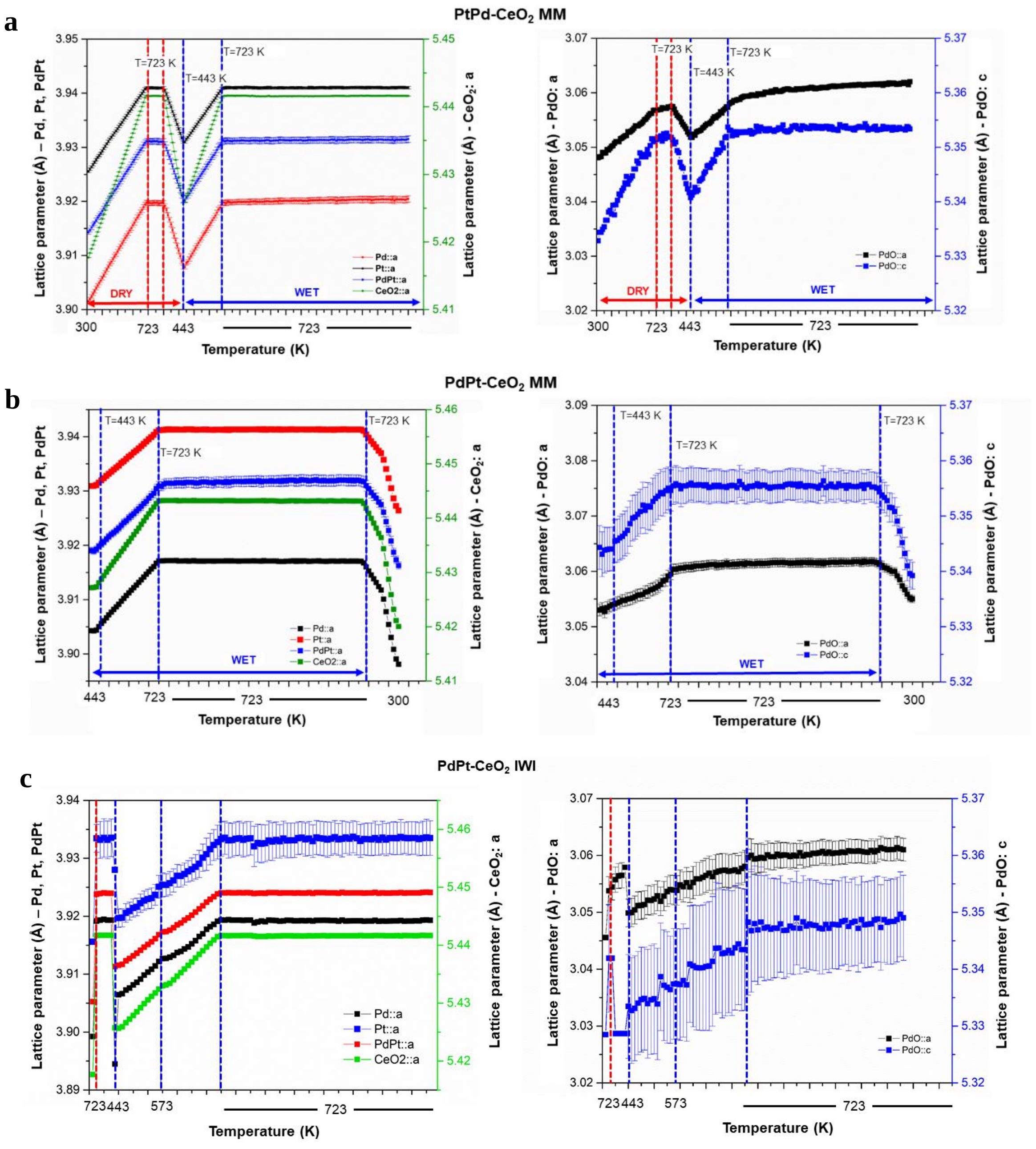

Figure S8. Lattice parameter obtained from the sequential Rietveld refinement of the diffractograms acquired during wet methane combustion for the bimetallic catalysts. **a**; PtPd-CeO_2 MM, **b**; PdPt-CeO_2 MM, **c**; PdPt/CeO_2 IWI. The error bars correspond to the standard deviation. MM: mechanical milling; IWI: incipient wetness impregnation.

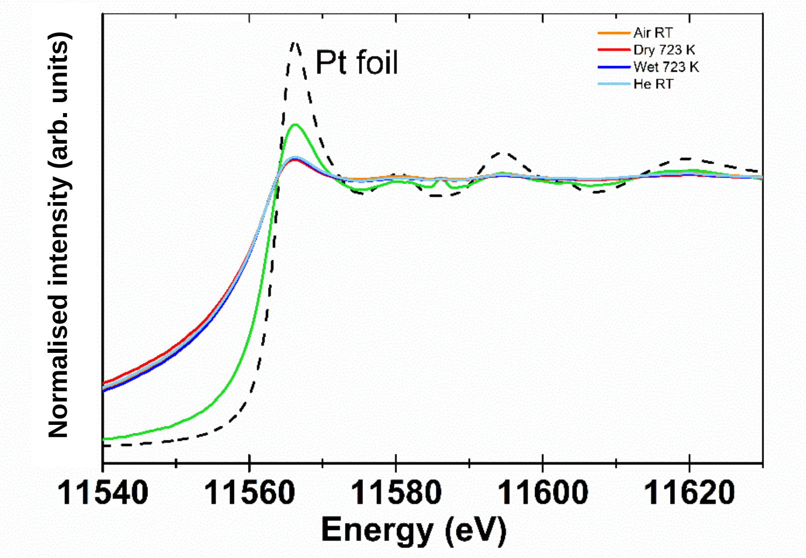

Figure S9. ***Operando*** **Pt L_3-edge XANES spectra of PtPd-CeO_2 MM after the TPC1173 treatment during dry and wet lean methane combustion.** TPC: Temperature-programmed combustion. MM: mechanical milling.

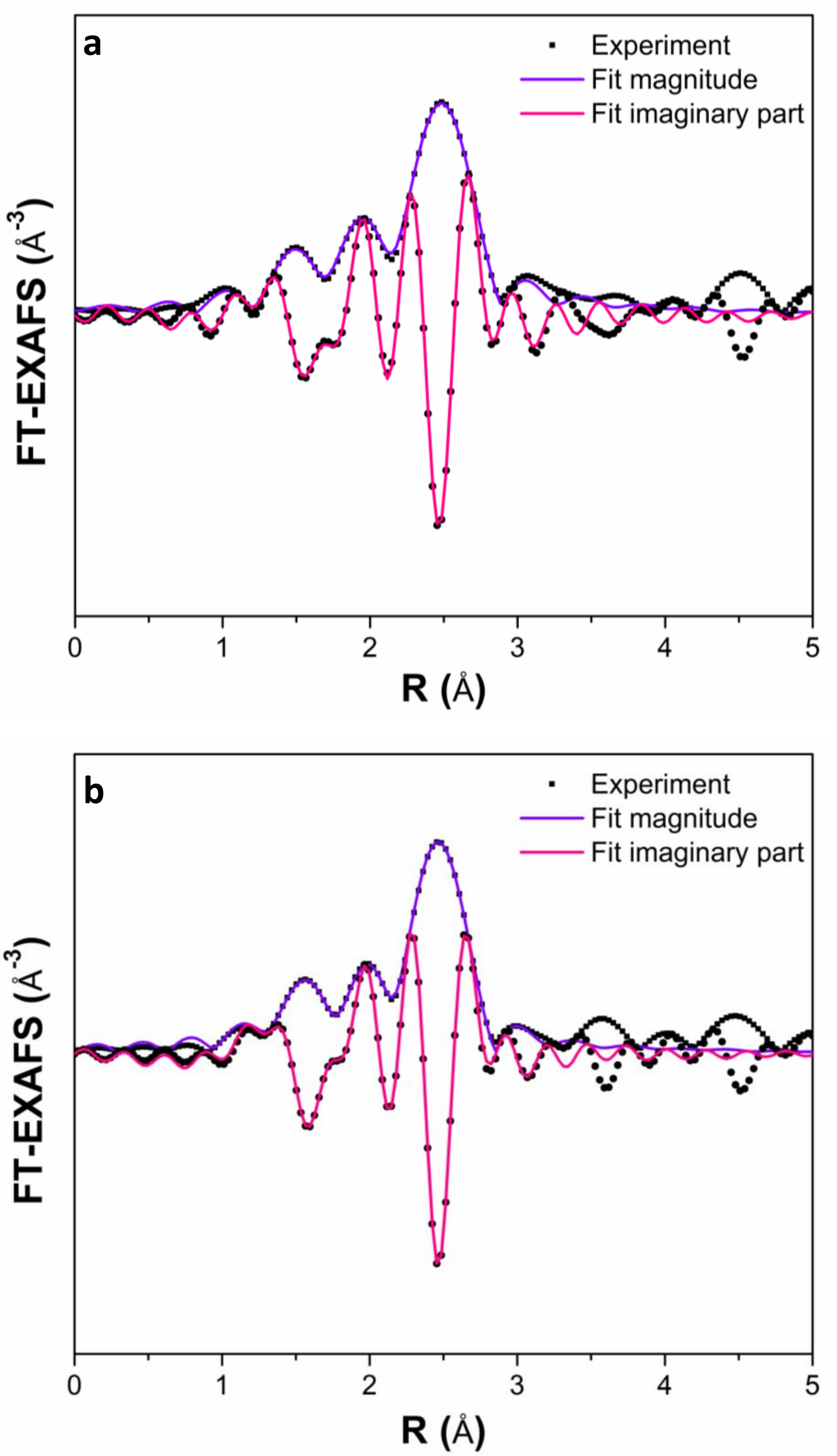

Figure S10. Fits of Pd K-edge EXAFS spectra. a, Pd-CeO_2 MM and **b,** PtPd-CeO_2 MM catalysts**.** The fitted spectra were acquired at room. Magnitudes and imaginary parts of FT-EXAFS spectra are shown. The fit was performed in R range: 1.3 Å to 3.3 Å. MM: mechanical milling.

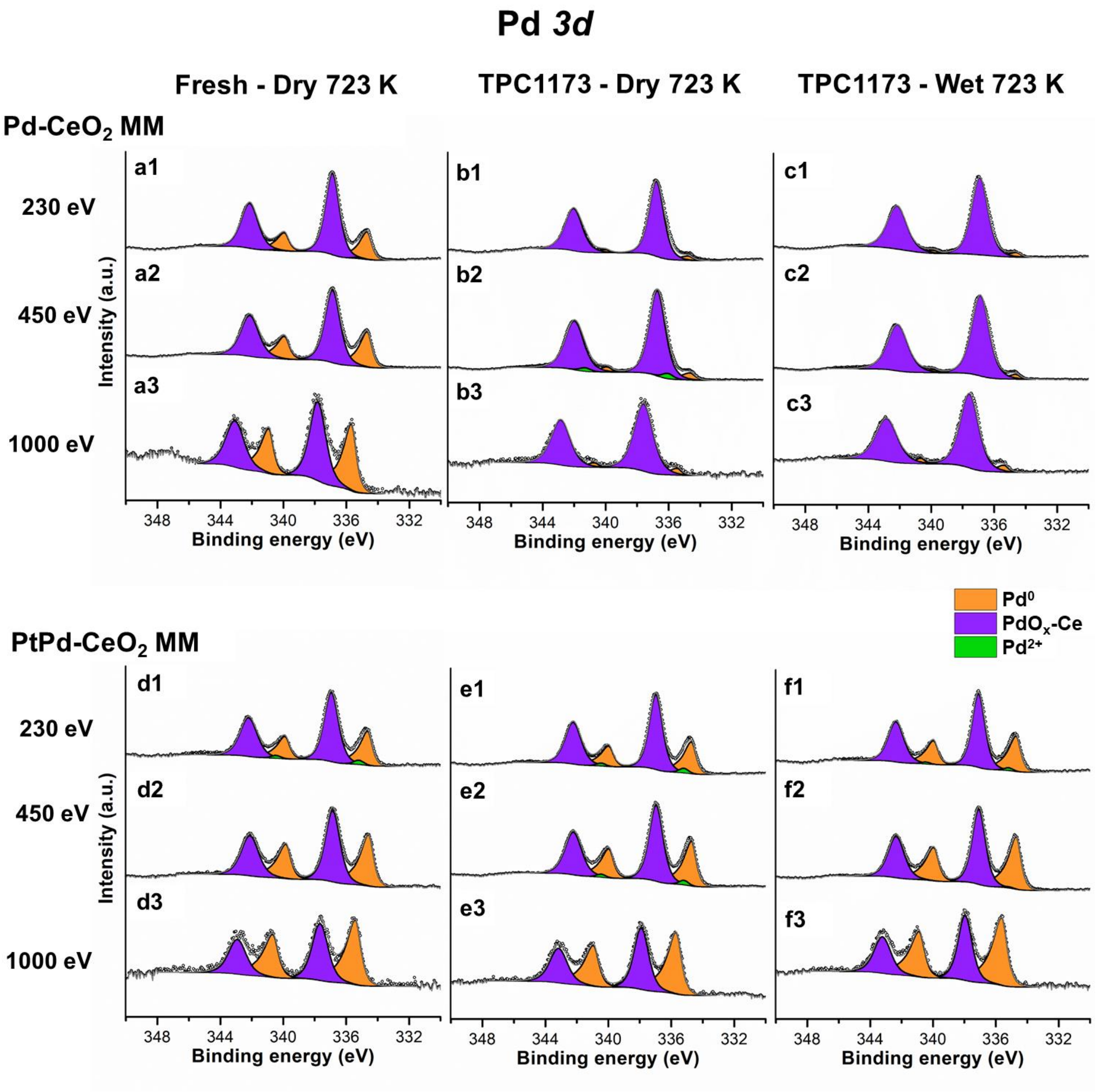

Figure S11. AP-XP spectra of the Pd *3d* region of Pd-CeO_2 MM and PtPd-CeO_2 MM catalysts. (**a-c**) correspond to Pd-CeO_2 MM and (**d-f**) to PtPd-CeO_2 MM catalysts, acquired at 230, 450, and 1000 eV kinetic energies under dry (**a** and **d**) and wet (**b-c** and **e-f**) methane combustion conditions at 723 K. The labels indicate the pretreatment and the gaseous atmosphere and temperature tested during the measurements. TPC: Temperature-programmed combustion. MM: mechanical milling.

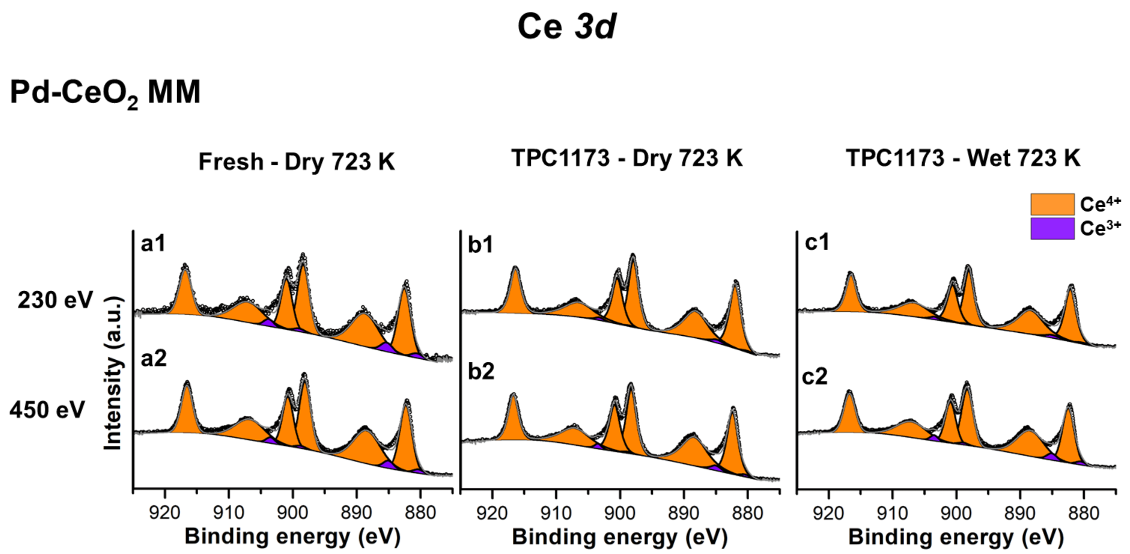

Figure S12. A**P-XP spectra of the Ce *3d* region of Pd-CeO_2 MM acquired at different kinetic energies**. The spectra were acquired during (**a1** to **b2**) dry and (**c1** and **c2**) wet methane combustion at 723 K. The labels indicate the pretreatment and the gaseous atmosphere and temperature tested during the measurements. TPC: Temperature-programmed combustion. MM: mechanical milling.

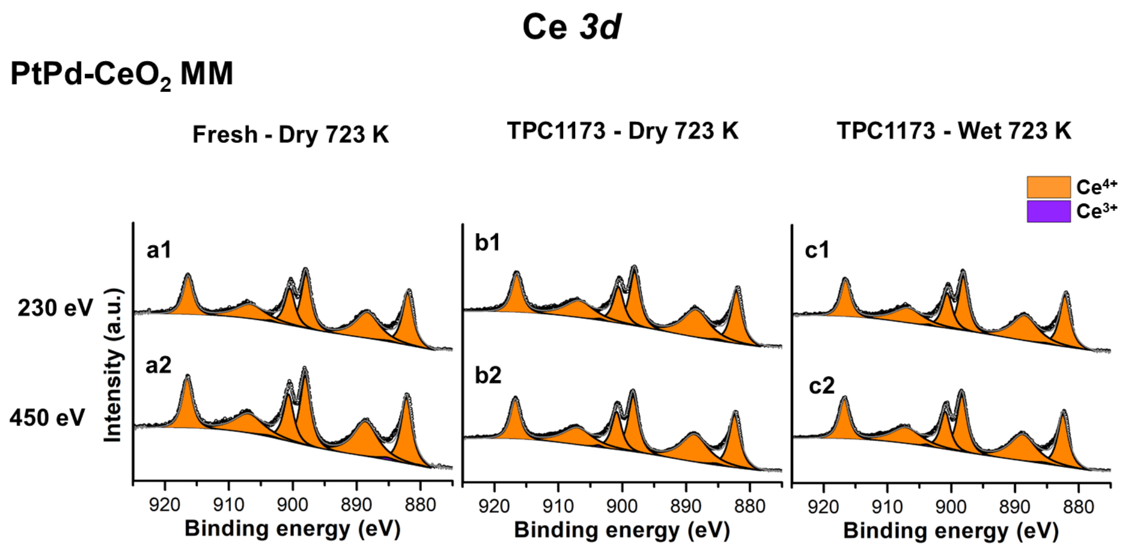

Figure S13. AP-XP spectra of the Ce *3d* region of PtPd-CeO_2 MM acquired at different kinetic energies. The spectra were acquired during (**a1** to **b2**) dry and (**c1** and **c2**) wet methane combustion at 723 K. The labels indicate the pretreatment and the gaseous atmosphere and temperature tested during the measurements. TPC: Temperature-programmed combustion. MM: mechanical milling.

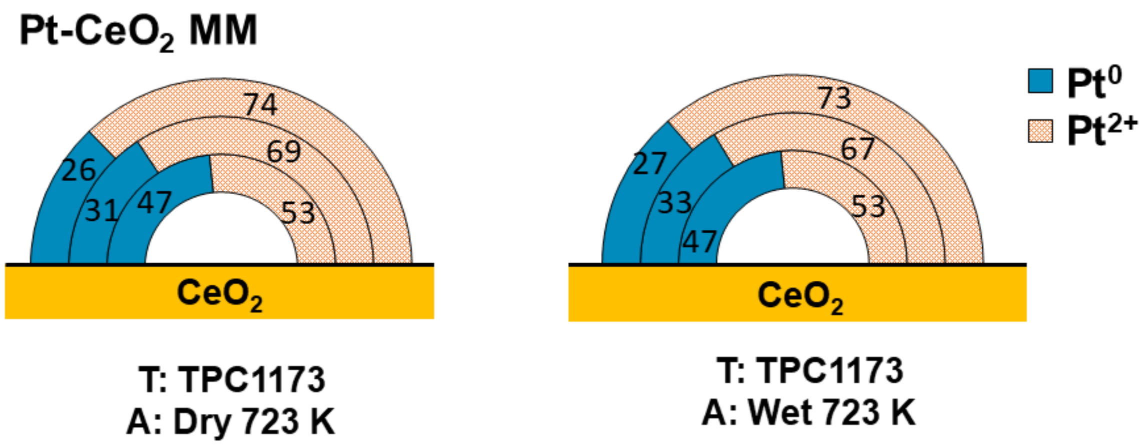

Figure S14. Platinum atomic fractions and oxidation states calculated for Pt-CeO_2 MM. The different regions shown in the semicircles correspond to different oxidation states as stated in the legend. The outer semicircle corresponds to the atomic fractions obtained with KE = 230 eV, the intermediate semicircle corresponds to KE = 450 eV, and the inner semicircle corresponds to KE = 1000 eV. ***T*** indicates the previous pretreatment performed to the catalyst. ***A*** indicates the gaseous atmosphere present during the measurements. MM: mechanical milling.

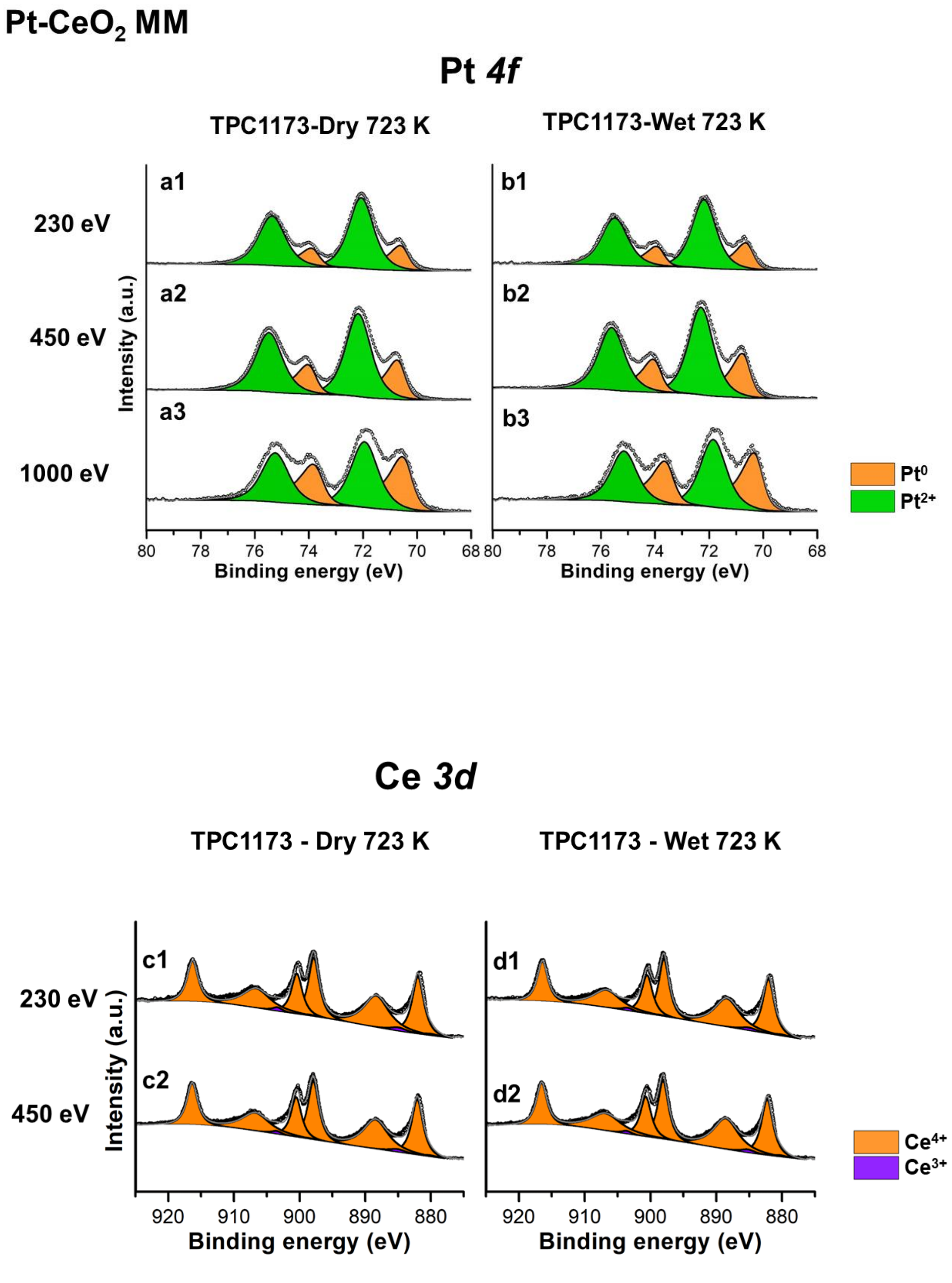

Figure S15. AP-XP spectra of Pt-CeO_2 MM after the TPC1173 treatment. The spectra were acquired at different kinetic energies during dry and wet methane combustion at 723 K. **a1** to **b3** correspond to the Pt *4f* region, **c1** to **d2**; to the Ce *3d* region. MM: mechanical milling.

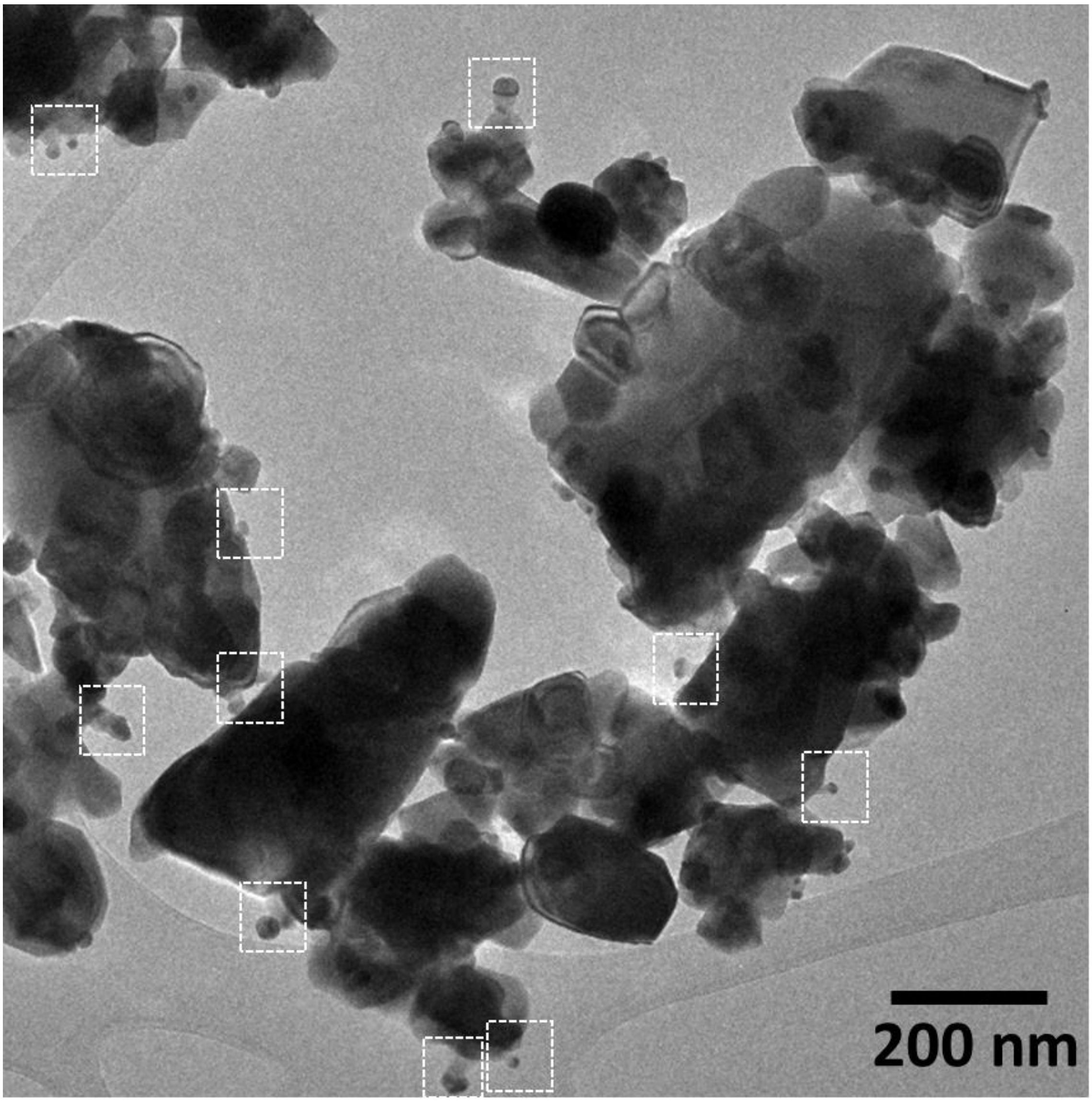

Figure S16. Low-magnification TEM image of a PtPd-CeO_2 MM catalyst after 24 h under TOS wet at 723 K. Mushroom-like structures identified on the ceria surface of a PtPd-CeO_2 MM catalyst after 24 h under TOS (time-on-stream) wet at 723 K. The squares enclose the mushroom structures identified. MM: mechanical milling.
